# Impaired Iron–Sulfur Cluster Synthesis Induces Mitochondrial PARthanatos in Diabetic Cardiomyopathy

**DOI:** 10.1002/advs.202406695

**Published:** 2024-11-04

**Authors:** Mengyi Wang, Shiwu Zhang, Jinwei Tian, Fan Yang, He Chen, Shuzhi Bai, Jiaxin Kang, Kemiao Pang, Jiayi Huang, Mingjie Dong, Shiyun Dong, Zhen Tian, Shaohong Fang, Huitao Fan, Fanghao Lu, Bo Yu, Shuijie Li, Weihua Zhang

**Affiliations:** ^1^ Department of Cardiology Second Affiliated Hospital of Harbin Medical University No. 246 Xuefu ROAD Harbin 150086 China; ^2^ Heilongjiang Provincial Key Laboratory of Panvascular Disease Harbin 150000 China; ^3^ Department of Pathophysiology Harbin Medical University Harbin 150000 China; ^4^ Key Laboratory of Myocardial Ischemia Ministry of Education Harbin 150000 China; ^5^ Department of Forensic Medicine Harbin Medical University Harbin 150000 China; ^6^ College of Bioinformatics Science and Technology Harbin Medical University Harbin 150000 China; ^7^ Department of Critical Care Medicine The First Affiliated Hospital of Harbin Medical University Harbin 150001 China; ^8^ Department of Hematology The First Affiliated Hospital of Harbin Medical University Harbin 150001 China; ^9^ NHC Key Laboratory of Cell Transplantation The First Affiliated Hospital of Harbin Medical University Harbin 150001 China; ^10^ Key Laboratory of Hepatosplenic Surgery of Ministry of Education The First Affiliated Hospital of Harbin Medical University Harbin 150001 China; ^11^ State Key Laboratory of Frigid Zone Cardiovascular Diseases (SKLFZCD) Harbin 150000 China; ^12^ State Key Laboratory of Frigid Zone Cardiovascular Diseases (SKLFZCD) Department of Biopharmaceutical Sciences College of Pharmacy Harbin Medical University Harbin 150000 China; ^13^ Heilongjiang Province Key Laboratory of Research on Molecular Targeted Anti‐Tumor Drugs Harbin 150000 China

**Keywords:** cysteine desulfurase (NFS1), diabetic cardiomyopathy (DCM), hydrogen sulfide (H_2_S), iron–sulfur (Fe–S) cluster, PARthanatos

## Abstract

Diabetic cardiomyopathy (DCM), a severe complication of diabetes, is characterized by mitochondrial dysfunction, oxidative stress, and DNA damage. Despite its severity, the intrinsic factors governing cardiomyocyte damage in DCM remain unclear. It is hypothesized that impaired iron–sulfur (Fe–S) cluster synthesis plays a crucial role in the pathogenesis of DCM. Reduced S‐sulfhydration of cysteine desulfurase (NFS1) is a novel mechanism that contributes to mitochondrial dysfunction and PARthanatos in DCM. Mechanistically, hydrogen sulfide (H_2_S) supplementation restores NFS1 S‐sulfhydration at cysteine 383 residue, thereby enhancing Fe–S cluster synthesis, improving mitochondrial function, increasing cardiomyocyte viability, and alleviating cardiac damage. This study provides novel insights into the interplay between Fe–S clusters, mitochondrial dysfunction, and PARthanatos, highlighting a promising therapeutic target for DCM and paving the way for potential clinical interventions to improve patient outcomes.

## Graphical abstract

A reduction in mitochondrial iron‐sulfur (Fe–S) cluster synthesis in diabetic cardiomyocytes decreases mitochondrial respiratory chain function, leading to oxidative stress and DNA damage. Supplementing with hydrogen sulfide (H_2_S) enhances Fe–S cluster synthesis via S‐sulfhydration of cysteine desulfurase (NFS1) at C383, preserving mitochondrial and nuclear DNA integrity and ameliorating diabetic cardiomyopathy (DCM).

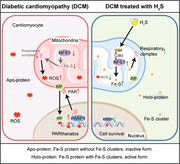

## 1 Introduction

Type 2 diabetes mellitus (T2DM) is a chronic metabolic disorder characterized by insulin resistance or relative insulin deficiency, which leads to elevated blood glucose and lipid levels, and subsequent systemic organ dysfunction. Diabetic cardiomyopathy (DCM) is a serious complication associated with T2DM. DCM is characterized by the prolonged dysregulation of glucose and lipid metabolism that induces sustained structural and functional heart damage, thereby significantly increasing the risk of cardiovascular diseases.^[^ ^1^, ^2^, ^3^, ^4^ ^]^

Recent evidence has linked DCM development to mitochondrial dysfunction and elevated intracellular reactive oxygen species (ROS) levels.^[^ ^5^, ^6^, ^7^, ^8^ ^]^ Defective mitochondrial respiratory chain complexes disrupt electron transfer, causing electron leakage and ROS generation, particularly superoxide^[^ ^9^, ^10^ ^]^ Furthermore, diabetes increases mitochondrial criticality, rendering the cardiac mitochondrial network highly susceptible to minor disturbances under intense oxidative stress. This increased sensitivity can lead to depolarization and synchronized oscillations throughout the myocardial syncytium.^[^ ^11^ ^]^ Disturbances in mitochondrial membrane potential (MMP) further exacerbate ROS formation, whereas the diminished antioxidant capacity of DCM cardiomyocytes amplifies ROS accumulation, resulting in extensive oxidative damage to proteins, DNA, and lipid membranes, thereby intensifying oxidative stress.^[^ ^12^, ^13^, ^14^ ^]^

Oxidative damage to the nuclear DNA disrupts normal base pairing, resulting in genetic mutations.^[^ ^15^ ^]^ Poly ADP‐ribose polymerase 1 (PARP1) plays a crucial role in DNA repair by catalyzing the generation of poly ADP‐ribose (PAR) to recruit other repair enzymes.^[^ ^16^, ^17^ ^]^ However, excessive PARP1 expression and activation lead to PAR accumulation, inducing translocation of mitochondrial apoptosis‐inducing factor (AIF) to the nucleus and causing DNA fragmentation‐mediated cell death, a process known as PARthanatos.^[^ ^18^, ^19^, ^20^, ^21^ ^]^

Iron–sulfur (Fe–S) clusters are crucial protein cofactors involved in mitochondrial respiration, citrate cycle, lipid metabolism, and DNA damage repair.^[^ ^22^, ^23^, ^24^, ^25^, ^26^, ^27^ ^]^ In the mitochondrial matrix, Fe–S clusters are synthesized by the Fe–S cluster assembly complex, with cysteine desulfurase (NFS1), Fe–S cluster assembly scaffold protein (ISCU), and frataxin (FXN) as core components. By using its conserved mobile S‐transfer loop to extract sulfur from cysteine, NFS1 provides an essential sulfur source for Fe–S cluster synthesis. ISCU accepts sulfur from NFS1 in the form of an S‐sulfhydrated intermediate and then binds iron ions to form the initial Fe–S clusters. FXN regulates iron supply and modulates NFS1 activity. The coordinated action of these proteins ensures the efficient synthesis of Fe–S clusters.^[^ ^28^, ^29^, ^30^, ^31^, ^32^ ^]^ Fe–S cluster depletion and oxidative stress contribute to the degradation of Fe–S proteins.^[^ ^33^ ^]^

Hydrogen sulfide (H_2_S), a gasotransmitter with cardiovascular protective properties, primarily regulates target proteins via S‐sulfhydration.^[^ ^34^, ^35^, ^36^, ^37^ ^]^ Previous studies indicated that insufficient endogenous H_2_S in DCM exacerbates cardiac damage.^[^ ^38^, ^39^ ^]^ Although exogenous H_2_S supplementation alleviates cardiac damage, the specific mechanisms and interplay between H_2_S and mitochondria are not fully understood. H_2_S contributes to electron generation via oxidation by sulfide quinone oxidoreductase (SQR), facilitating electron transport in the mitochondrial respiratory chain and subsequent ATP synthesis.^[^ ^40^ ^]^ Despite these findings, the precise mechanisms by which H_2_S influences mitochondrial function in DCM, particularly regarding its role in Fe–S cluster synthesis and mitochondrial respiration, remain unclear. This gap in our understanding hinders the development of targeted therapies to mitigate mitochondrial dysfunction and oxidative stress in DCM.

To address this issue, we utilized leptin receptor gene knockout (db/db) mice, a well‐established model of T2DM, and primary cardiomyocytes to investigate the molecular mechanisms underlying DCM. Our research uncovers a novel mechanism by which H_2_S maintains mitochondrial respiration and mitigates PARthanatos. We found that both the expression and S‐sulfhydration of NFS1 significantly decreased in diabetic cardiomyocytes, which impaired Fe–S cluster synthesis. Fe–S cluster deficiency disrupts mitochondrial respiration and leads to DNA damage‐induced PARthanatos. H_2_S supplementation enhances NFS1 S‐sulfhydration at cysteine 383 (C383), thereby promoting Fe–S cluster synthesis. Knockdown or mutation of NFS1 at C383 residue abrogates the protective effects of H_2_S. Thus, H_2_S ameliorates mitochondrial dysfunction and DNA damage in DCM by promoting Fe–S cluster synthesis. Given its role in maintaining mitochondrial function, NFS1 may represent a promising target for clinical strategies aimed at preventing or treating DCM, providing a new theoretical foundation for disease intervention.

## 2 Results

### 2.1 Cardiac Structure and Function are Impaired in Diabetic Mice

**Figure 1:**
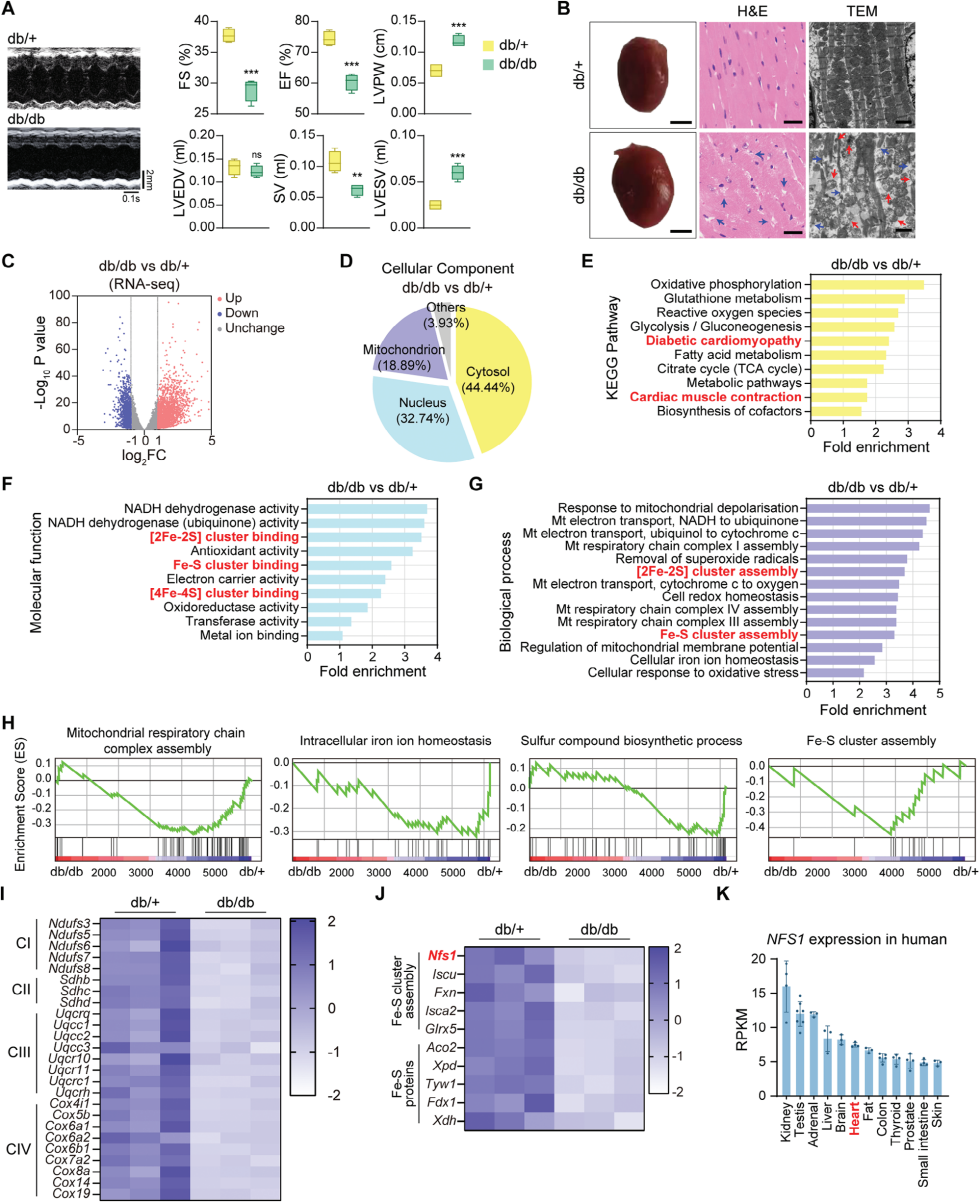
Cardiac structure and function are impaired in diabetic mice. A) Representative left ventricular M‐mode echocardiographic tracings and quantification of fractional shortening (FS) %, ejection fraction (EF) %, left ventricular posterior wall (LVPW), left ventricular end‐diastolic volume (LVEDV), stroke volume (SV) and left ventricular end‐systolic volume (LVESV) of mice (n = 4). B) Representative photographs of mouse hearts (scale bars, 2 mm), H&E staining sections of the left ventricle (scale bars, 25 µm), and TEM images of left ventricular sections (scale bars, 2 µm). The blue arrows indicate abnormal cardiac structures. The red arrows indicate damaged mitochondria. C) Volcano plot of DEGs between db/db and db/+ mouse hearts acquired from RNA‐Seq. D) Pie chart representing the results of cellular component enrichment analysis based on DEGs. E) KEGG pathway enrichment analysis of DEGs. F) Molecular functions of GO enrichment of DEGs. G) Biological processes of GO enrichment of DEGs. H) GSEA of DEGs. I) Heatmap of DEGs involved in mitochondrial respiratory complexes. CI‐IV, complex I–IV. J) Heatmap of DEGs associated with Fe–S clusters. K) Tissue‐specificity of NFS1 in humans. All quantitative data are presented as mean ± SD from independent experiments. ^**^ *P* < 0.01, ^***^ *P* < 0.001 versus db/+ by unpaired *t* test.

To investigate cardiac dysfunction in diabetes, db/db mice were used as a diabetic model owing to their increased body weight, elevated blood glucose levels, and impaired glucose tolerance, which are typical manifestations of T2DM (Figures S1A,B, Supporting Information). Heterozygous littermates (db/+ mice) served as controls. Results from echocardiography revealed decreased cardiac contractile function in db/db mice. Specifically, db/db mice showed significant reductions in fractional shortening (FS)%, ejection fraction (EF)%, and stroke volume (SV) compared to db/+ mice. In addition, db/db mice exhibited increased left ventricular posterior wall (LVPW) thickness and left ventricular end‐systolic volume (LVESV), with no significant changes in left ventricular end‐diastolic volume (LVEDV) (**Figure** **1**A). These findings indicate impaired cardiac contractility in diabetic mice. The heart‐to‐tibia ratio was significantly higher in db/db mice than the heart‐to‐tibia ratio in db/+ mice, indicating abnormal cardiac enlargement (Figure S1C, Supporting Information). Hematoxylin and eosin (H&E) staining and transmission electron microscopy (TEM) revealed that the cardiac tissues of db/db mice displayed a disorganized arrangement, partial disruption, mitochondrial fragmentation, and abnormal alterations in mitochondrial cristae structure (Figure 1B).

To elucidate the molecular mechanisms responsible for cardiac damage in diabetes, we conducted a comprehensive analysis of the cardiac transcriptomes of db/db mice and db/+ mice (Figure 1C). A substantial proportion of proteins encoded by the differentially expressed genes (DEGs) between these two groups (18.89%) were localized in the mitochondria (Figure 1D). Kyoto Encyclopedia of Genes and Genomes (KEGG) pathway analysis revealed significant enrichment of DEGs in pathways associated with DCM and cardiac muscle contraction (Figure 1E). Gene Ontology (GO) and Gene Set Enrichment Analyses (GSEA) showed significant enrichment of genes related to the mitochondrial respiratory chain, iron ion homeostasis, sulfur compound biosynthesis, and Fe–S cluster binding/assembly (Figure 1F–H).

To further confirm the expression changes in Fe–S cluster‐related genes in diabetic hearts, we analyzed single‐cell RNA sequencing data (GSE213337) of cardiac specimens collected from healthy mice (Ctrl) and mice with diabetes mellitus (DM) from the public Gene Expression Omnibus (GEO) database.^[^ ^41^ ^]^ Analysis of DEGs in cardiomyocyte subpopulations revealed enrichment of glucose/lipid metabolism, mitochondrial oxidative phosphorylation, (ROS metabolic processes, and Fe–S cluster biosynthesis (Figure S1D, Supporting Information). Furthermore, GSEA indicated pronounced enrichment of DEGs in pathways involving mitochondrial electron transport and respiratory chain complex assembly (Figure S1E, Supporting Information). Notably, the downregulated DEGs induced the genes responsible for encoding proteins associated with mitochondrial respiration (Figure S1F, Supporting Information). These observations further confirm that Fe–S cluster synthesis disruption and mitochondrial respiratory dysfunction occur in the cardiomyocytes of diabetic mice.

Among the genes associated with the mitochondrial respiratory chain, multiple genes involved in the formation of complexes I, II, III, and IV were downregulated in the hearts of db/db mice (Figure 1I). Furthermore, among the genes involved in de novo synthesis of Fe–S clusters, *Nfs1*, *Iscu*, and frataxin (*Fxn*) exhibited reduced expression in db/db mice (Figure 1J). NFS1 is a highly conserved protein abundantly expressed in the human heart and other parenchymal organs (Figure 1K). Taken together, these data suggest that mitochondrial dysfunction, especially damage to the Fe–S cluster assembly, emerges in DCM.

### 2.2 Disruptions of Fe–S Cluster Synthesis in Diabetic Cardiomyopathy

**Figure 2:**
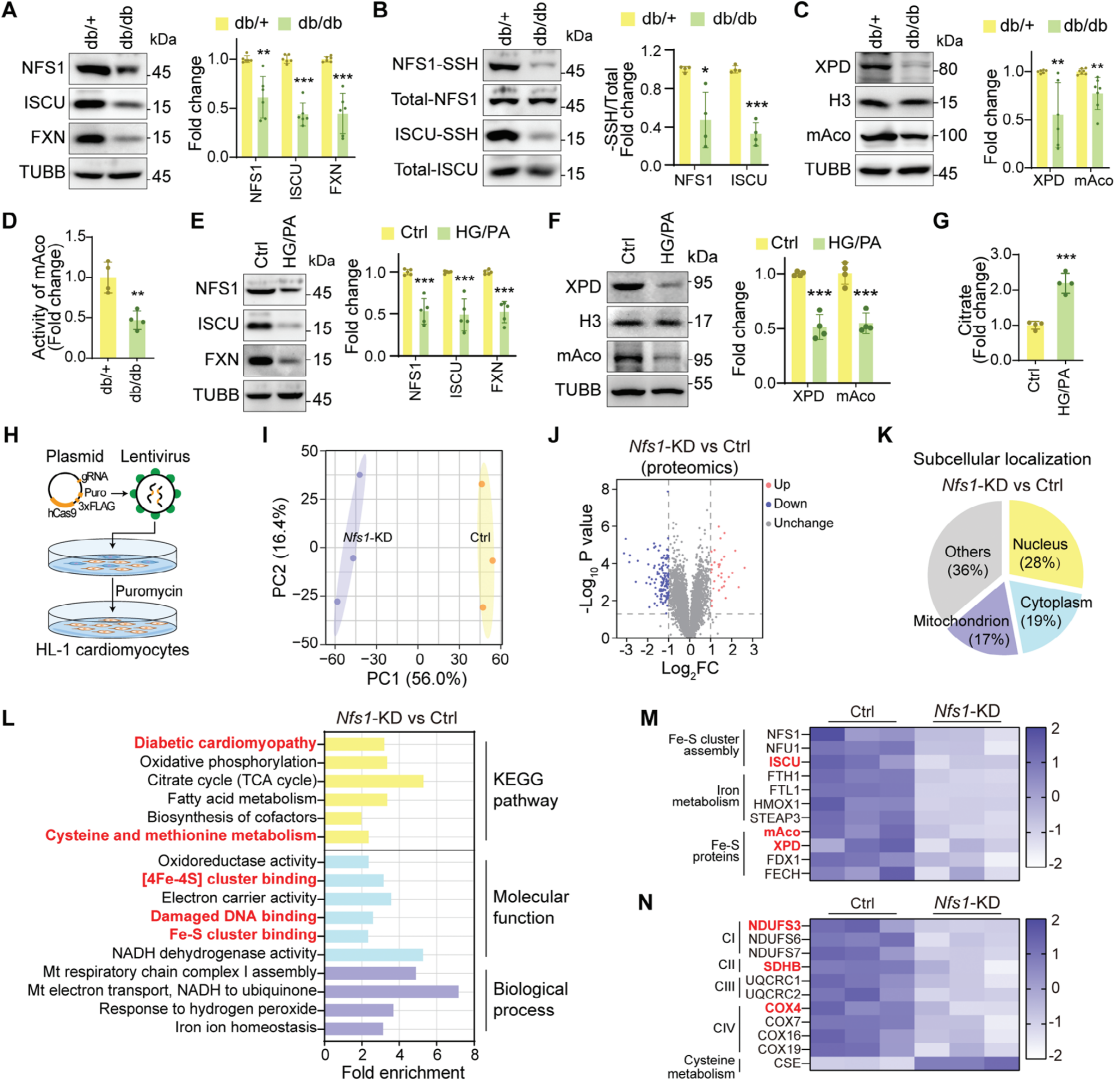
Disruptions of Fe–S cluster synthesis in diabetic cardiomyopathy. A) Protein levels of NFS1, ISCU, and FXN in cardiac tissues (n = 6). B) S‐sulfhydration levels of NFS1 and ISCU in cardiac tissues (n = 4). C) Protein levels of XPD (n = 6) and mAco (n = 7) in cardiac tissues. D) Activity of mAco in cardiac tissues (n = 4). E) Protein levels of NFS1, ISCU, and FXN in cardiomyocytes (n = 5). F) Protein levels of XPD and mAco in cardiomyocytes (n = 4). G) Citrate concentration in cardiomyocytes (n = 4). H) Workflow for *Nfs1*‐KD cardiomyocyte cell line establishment. I) Principal component analysis (PCA) plot of *Nfs1*‐KD and control groups. J) Volcano plot of DEPs in the proteomic analysis. K) Subcellular localization of the top 100 DEPs. L) Enrichment of molecular functions, biological processes, and KEGG pathways of DEPs. M) Heatmap of proteins related to Fe–S clusters and iron homeostasis. N) Heatmap of proteins related to the mitochondrial respiratory chain and cysteine metabolism. All quantitative data are presented as mean ± SD from independent experiments. ^*^ *P* < 0.05, ^**^ *P* < 0.01, ^***^ *P* < 0.001 versus db/+ or Ctrl by unpaired *t* test.

To understand how diabetes affects Fe–S cluster synthesis, we examined the key proteins involved in this process. NFS1 transfers sulfur to ISCU through the formation of S‐sulfhydrated intermediates, facilitating Fe–S cluster synthesis.^[^ ^32^ ^]^ We observed significantly reduced NFS1, ISCU, and FXN protein levels in db/db mice (**Figure** **2**A). To visualize the S‐sulfhydration levels of these target proteins, we used biotin switch assays, which exploit the affinity of biotin for free thiol groups (Figure S2A, Supporting Information). The findings of this method revealed significantly reduced S‐sulfhydration levels of NFS1 and ISCU in db/db mice (Figure 2B). Mitochondrial aconitase (mAco/Aco2) enzyme involved in the citrate cycle and Xeroderma pigmentosum Group D (XPD) protein involved in nuclear DNA repair contain Fe–S clusters.^[^ ^22^, ^42^ ^]^ In our study, db/db mice showed reduced XPD expression along with decreased protein levels and mAco activity, indicating the loss of Fe–S clusters (Figure 2C,D).

To mimic the hyperglycemic and hyperlipidemic environment in vitro, primary neonatal mouse cardiomyocytes were exposed to high glucose (HG, 40 mm) and palmitate (PA, 200 µm). This treatment led to similar alterations in Fe–S cluster assembly proteins and Fe–S proteins as observed in vivo (Figure 2E,F). Additionally, the accumulation of citrate, a substrate for mAco‐mediated enzymatic reactions, indicated the loss of mAco activity in the HG/PA‐treated groups (Figure 2G). Furthermore, we found a significant accumulation of intracellular iron ions in HG/PA‐treated cardiomyocytes, which resulted from decreased intracellular iron utilization (Figure S2B, Supporting Information). These findings suggest a reduction in Fe–S cluster synthesis in diabetic cardiomyocytes.

To further explore the role of impaired Fe–S cluster synthesis in DCM, we employed CRISPR/Cas9 gene editing to knock down the Nfs1 gene (*Nfs1*‐KD) in cardiomyocytes. Of the three knockdown strategies tested, Y28627 exhibited the most stable effects (Figure S2C, Supporting Information). We established an *Nfs1*‐KD cardiomyocyte cell line and performed proteomic analyses (Figure 2H,I). The results revealed that NFS1 depletion led to significant alterations in the expression profiles of 668 proteins, with 217 upregulated and 451 downregulated proteins (Figure 2J). Notably, ≈17% of the top 100 differentially expressed proteins (DEPs) were localized within the mitochondria (Figure 2K). Functional enrichment analysis showed that the depletion of NFS1 affected several physiological processes, including diabetic cardiomyopathy (including alterations in energy metabolism, mitochondrial dysfunction, and cardiac structural changes), cysteine/methionine metabolism, Fe–S cluster binding and damaged DNA binding (Figure 2L). Moreover, we observed that the knockdown of Nfs1 led to the downregulation of proteins involved in Fe–S cluster biosynthesis and the mitochondrial respiratory chain (Figure 2M,N). These results suggest a potential link between disruptions in Fe–S cluster synthesis and the development of DCM, as well as mitochondrial and DNA damage.

### 2.3 Impaired Fe–S Cluster Synthesis Induces Mitochondrial PARthanatos and Cardiomyocyte Death in Diabetic Cardiomyopathy

**Figure 3:**
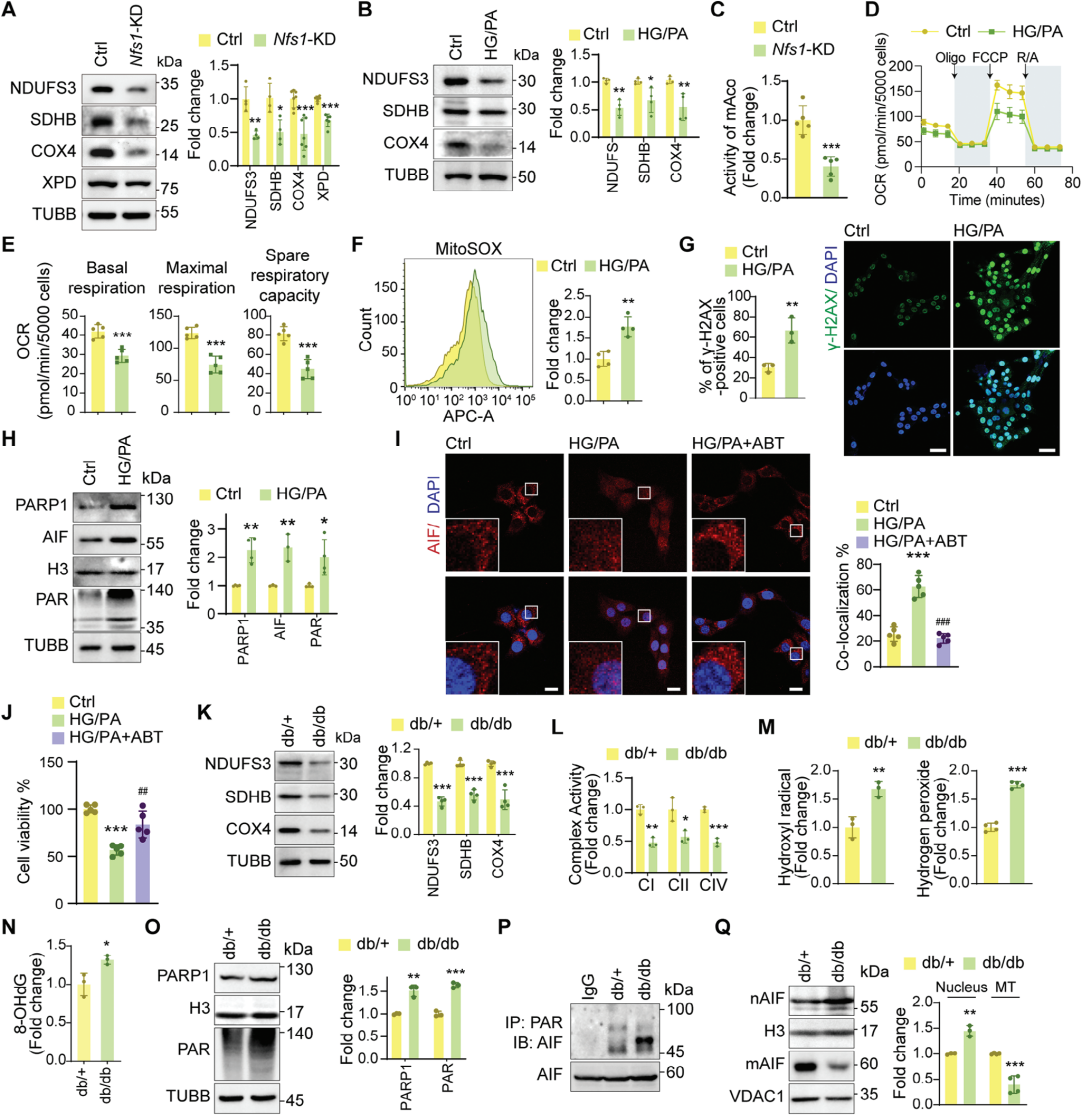
Impaired Fe–S cluster synthesis induces mitochondrial PARthanatos and cardiomyocyte death in diabetic cardiomyopathy. A) Protein levels of NDUFS3 (n = 4), SDHB (n = 4), COX4 (n = 7), and XPD (n = 6) in cardiomyocytes. B) Protein levels of NDUFS3, SDHB, and COX4 in cardiomyocytes (n = 4). C) Activity of mAco in cardiomyocytes (n = 5). D) Normalized OCR of cardiomyocytes (n = 5). E) Quantification of OCR in D (n = 5). F) Flow cytometry results showing MitoSOX staining of cardiomyocytes (n = 4). G) γ‐H2AX foci in cardiomyocytes (n = 3). Scale bars, 50 µm. H) Protein levels of PARP1 (n = 4), AIF (n = 3), and PAR (n = 4) in cardiomyocytes. I) Confocal microscopy showing AIF (red) co‐localization with nuclei (blue) in cardiomyocytes (n = 5). Scale bars, 20 µm. J) Viability of cardiomyocytes (n = 5). K) Protein levels of NDUFS3, SDHB, and COX4 in cardiac tissues (n = 4). L) Activity of mitochondrial complexes I, II, and IV in cardiac tissues (n = 3). M) Concentrations of hydroxyl radicals (n = 3) and hydrogen peroxide (n = 4) in cardiac tissues. N) Quantification of 8‐OHdG levels in cardiac tissues (n = 3). O) PARP1 and PAR protein levels in cardiac tissues (n = 3). P) Interaction between PAR and AIF in cardiac tissues. Q) Protein levels of nuclear AIF (nAIF, n = 3) and mitochondrial AIF (mAIF, n = 4) in cardiac tissues. MT, mitochondrion. All quantitative data are presented as mean ± SD from independent experiments. ^*^ *P* < 0.05, ^**^ *P* < 0.01, ^***^ *P* < 0.001 versus Ctrl or db/+; ^##^ *P* < 0.01, ^###^ *P* < 0.001 versus HG/PA by unpaired *t* ‐test or ordinary one‐way ANOVA.

Fe–S clusters are critical components of mitochondrial respiratory complexes, such as NDUFS1/2/3 in complex I (NADH ubiquinone oxidoreductase) and SDHB in complex II (succinate dehydrogenase).^[^ ^43^, ^44^, ^45^ ^]^ In *Nfs1*‐KD group, the protein levels of NDUFS3, SDHB, and COX4 (complex IV) were diminished, consistent with the proteomic analysis results (**Figure** **3**A), implying that NFS1 deficiency impairs mitochondrial respiration. Similar reductions in the levels of these respiratory complex proteins were observed in HG/PA‐treated cardiomyocytes (Figure 3B). Additionally, *Nfs1*‐KD led to decreased mAco activity, which is consistent with the reduction observed in cardiac tissues of db/db mice, as shown in Figure 2D (Figure 3C).

To further investigate mitochondrial function, we monitored changes in mitochondrial oxygen consumption rate (OCR) in cardiomyocytes. HG/PA treatment disrupted mitochondrial respiration, as evidenced by impaired basal respiration, maximal respiration, and spare respiratory capacity (Figure 3D,E). The mitochondrial respiratory chain is the primary source of ROS, that cause cellular oxidative stress. ROS are a general term for a large family of oxidants and mainly include hydroxyl radicals, hydrogen peroxide, and superoxide.^[^ ^46^ ^]^ Mitochondria‐derived ROS (superoxide) were detected using MitoSOX probes and flow cytometry, which revealed a significant increase in mitochondrial ROS levels in cardiomyocytes treated with HG/PA (Figure 3F). Direct measurement revealed a significant increase in superoxide levels in the HG/PA‐treated group (Figure S2D, Supporting Information). Analysis of dihydroethidium (DHE) staining confirmed elevated cellular ROS levels in cardiomyocytes treated with HG/PA (Figure S2E, Supporting Information). The analysis of Tetramethyl‐ rhodamine ethyl ester (TMRE) staining revealed demonstrated mitochondrial membrane potential (MMP) depolarization in the HG/PA‐treated groups, indicating abnormal electron transfer in the mitochondria (Figure S2F, Supporting Information).

XPD, a DNA helicase essential for nucleotide excision repair, was downregulated in *Nfs1*‐KD groups (Figure 3A). As a biomarker of DNA damage, the percentage of γ‐H2AX‐positive cardiomyocytes was significantly higher in HG/PA‐treated group, indicating compromised nuclear DNA integrity (Figure 3G). The initial step in the DNA damage repair response is the expression of poly ADP‐ribose polymerase (PARP1). Excessive poly ADP‐ribose (PAR) produced by PARP1 binds to mitochondrial apoptosis‐inducing factor (AIF), leading to its release and subsequent nuclear translocation, causing DNA fragmentation and cell death, in a process known as PARthanatos.^[^ ^17^, ^18^, ^19^, ^21^ ^]^ Nuclear PARP1 and AIF levels were significantly elevated in the HG/PA‐treated group, accompanied by PAR accumulation (Figure 3H). Confocal microscopy analysis revealed noticeable AIF nuclear translocation under HG/PA conditions, which could be reversed by the PARP1 inhibitor ABT888 (Figure 3I). Hyperglycemic and hyperlipidemic environments induced cardiomyocyte death, which was partially ameliorated by ABT888 treatment (Figure 3J).

The in vivo expression and activity of mitochondrial complexes were downregulated in db/db mice (Figure 3K,L). Hydroxyl radical and hydrogen peroxide levels were significantly increased in the db/db mice (Figure 3M). Moreover, there was a significant increase in the DNA oxidative damage marker 8‐hydroxy‐2′‐deoxyguanosine (8‐OHdG) levels in db/db mice (Figure 3N). These mice also exhibited PARP1 and PAR upregulation (Figure 3O). The findings of Co‐immunoprecipitation (Co‐IP) experiments confirmed the enhanced interaction between AIF and PAR in db/db mice (Figure 3P). These changes caused the translocation of AIF from the mitochondria to the nucleus (Figure 3Q). These results suggest that impaired Fe–S cluster synthesis leads to ROS accumulation and severe mitochondrial damage, inducing DNA damage and subsequent PARthanatos in diabetic cardiomyocytes.

### 2.4 H_2_S Supplementation Improves Cardiac Function in Diabetic Cardiomyopathy

**Figure 4:**
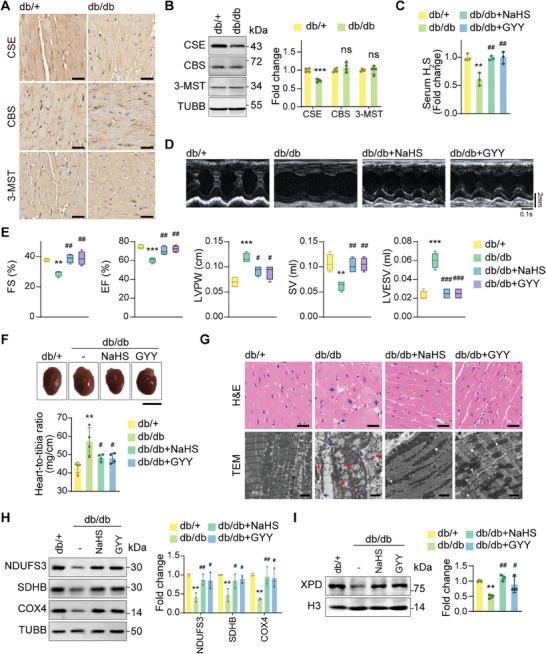
H_2_S supplementation improves cardiac function in diabetic cardiomyopathy. A) Representative immunohistochemistry (IHC) staining images of CSE, CBS, and 3‐MST in cardiac tissues. Scale bars, 25 µm. B) Protein levels of CSE, CBS, and 3‐MST in cardiac tissues (n = 4). C) Serum H_2_S levels in mice (n = 3). D) Representative M‐mode echocardiography images of mouse hearts. E) Quantification of FS%, EF%, LVPW, SV, and LVESV (n = 4). F) Representative images of the hearts and quantification of heart‐to‐tibia values in mice (n = 4). Scale bars, 5 mm. G) Representative photographs of H&E‐stained sections of the left ventricle (scale bars, 25 µm) and TEM images of left ventricular sections (scale bars, 2 µm). The blue arrows indicate abnormal cardiac structures. The red arrows indicate damaged mitochondria. H) Protein levels of NDUFS3, SDHB, and COX4 in cardiac tissues (n = 3). I) XPD protein levels in cardiac tissues (n = 3). All quantitative data are presented as mean ± SD from independent experiments. ^**^ *P* < 0.01, ^***^ *P* < 0.001 versus db/+; ^#^ *P* < 0.05, ^##^ *P* < 0.01, ^###^ *P* < 0.001 versus db/db by unpaired *t* test or ordinary one‐way ANOVA.

Sulfur compound biosynthesis and cysteine metabolism were found to be affected in the hearts of db/db mice and *Nfs1*‐KD group cardiomyocytes, respectively. Hydrogen sulfide (H_2_S) is an endogenous gaseous signaling molecule and an inorganic sulfur‐containing compound that modulates the stability or activity of proteins through S‐sulfhydration of cysteine residues.^[^ ^37^ ^]^ The primary enzymes responsible for endogenous H_2_S production are cystathionine γ‐lyase (CSE), cystathionine β‐synthase (CBS), and 3‐mercaptopyruvate sulfurtransferase (3‐MST).^[^ ^40^, ^47^ ^]^ Our results revealed a significant reduction in CSE expression in the cardiac tissues of db/db mice, whereas CBS and 3‐MST expression levels remained relatively unchanged (**Figure** **4**A,B; Figure S3A, Supporting Information). Correspondingly, serum H_2_S concentration was markedly decreased in db/db mice (Figure 4C).

To address the deficit in H_2_S, we treated diabetic models with two exogenous H_2_S donors: NaHS (a classic H_2_S donor) and GYY4137 (a slow‐releasing H_2_S donor). Intraperitoneal injections of NaHS or GYY4137 provided exogenous H_2_S supplementation to db/db mice, effectively increasing serum H_2_S levels (Figure 4C). Similar patterns of CSE, CBS, and 3‐MST expression were observed in vitro (Figure S3B, Supporting Information). A significant decrease in H_2_S levels was observed in the HG/PA‐treated group, which was restored by treatment with NaHS or GYY4137 (Figure S3C, Supporting Information).

In db/db mice, NaHS or GYY4137 treatment significantly improved cardiac function (Figure 4D). Enhanced echocardiographic parameters, including EF%, FS%, SV, LVPW, and LVESV, were observed in H_2_S‐treated db/db mice (Figure 4E). H_2_S supplementation also alleviated abnormal cardiac enlargement in these mice (Figure 4F). Histological analyses with H&E staining and TEM demonstrated that exogenous H2S alleviated the disordered arrangement of myocardial fibers and restored mitochondrial morphology in db/db mouse hearts (Figure 4G).

Furthermore, we examined the effects of H_2_S supplementation on Fe–S proteins. H_2_S treatment preserved the levels of key Fe–S proteins, including NDUFS3, SDHB, and XPD, in db/db mice (Figure 4H,I). Collectively, these findings suggest that H_2_S supplementation effectively enhances cardiac function and increases the Fe–S protein levels in db/db mice.

### 2.5 H_2_S Supplementation Abrogates Mitochondrial Dysfunction and PARthanatos in Diabetic Cardiomyocytes

**Figure 5:**
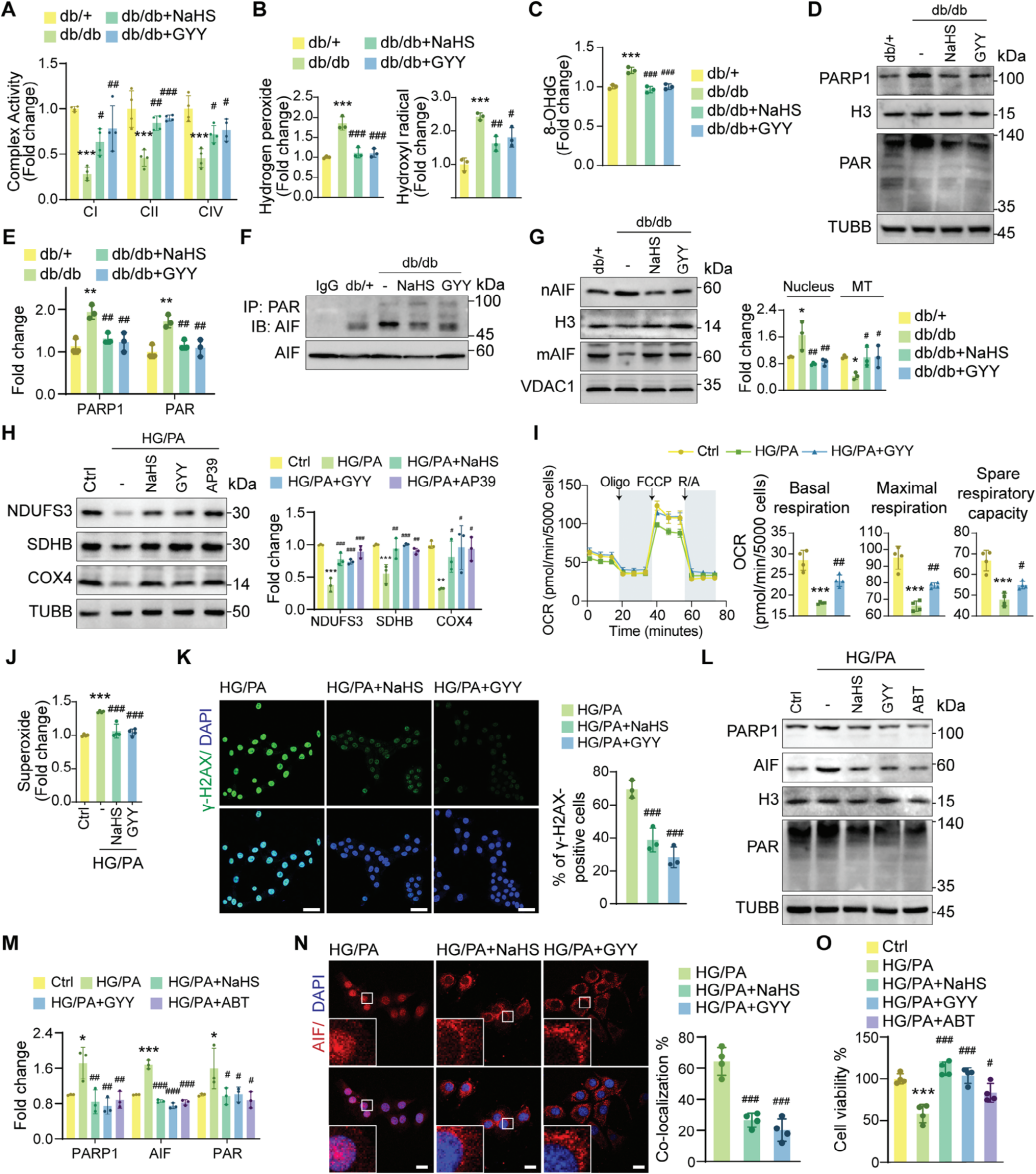
H_2_S supplementation abrogates mitochondrial dysfunction and PARthanatos in diabetic cardiomyocytes. A) Activity of mitochondrial complexes I, II, and IV in cardiac tissues (n = 4). B) Concentration of hydroxyl radicals and hydrogen peroxide in cardiac tissues (n = 3). C) Quantification of 8‐OHdG levels in cardiac tissues (n = 3). D) Protein levels of PARP1 and PAR in cardiac tissues. E) Quantification of (D) (n = 3). F) Interaction between PAR and AIF in cardiac tissues. G) Protein levels of nAIF and mAIF in cardiac tissues (n = 3). H) Protein levels of NDUFS3, SDHB, and COX4 in cardiomyocytes (n = 3). I) Normalized OCR of cardiomyocytes (n = 4). J) Superoxide concentration in cardiomyocytes (n = 4). K) γ‐H2AX foci in cardiomyocytes (n = 3). Scale bars, 50 µm. L) Protein levels of PARP1, AIF, and PAR in cardiomyocytes. M) Quantification of L (n = 3). N) Confocal microscopy showing AIF (red) co‐localization with nuclei (blue) in cardiomyocytes (n = 4). Scale bars, 20 µm. O) Viability of cardiomyocytes (n = 4). All quantitative data are presented as mean ± SD from independent experiments. ^*^ *P* < 0.05, ^**^ *P* < 0.01, ^***^ *P* < 0.001 versus db/+ or Ctrl; ^#^P < 0.05, ^##^P < 0.01, ^###^P < 0.001 versus db/db or HG/PA by ordinary one‐way ANOVA.

H_2_S is known for its role in reducing oxidative stress. In the present study, we investigated the effects of H_2_S supplementation on mitochondrial dysfunction and PARthanatos in diabetic cardiomyocytes. By elevating the levels of NDUFS3, SDHB, and COX4 proteins, exogenous H_2_S increased the activity of mitochondrial complexes in db/db mice (**Figure** **5**A). This was accompanied by a reduction in ROS accumulation in cardiac tissues, demonstrating the antioxidative properties of H_2_S (Figure 5B). Given its dual effects on XPD and ROS, H_2_S effectively reduced the levels of 8‐OHdG, a marker of oxidative DNA damage, in the hearts of db/db mice (Figure 5C). Further investigation revealed that H_2_S supplementation decreased PARP1 levels and PAR production (Figure 5D,F). Co‐IP analysis confirmed a decreased interaction between PAR and AIF in db/db mice treated with H_2_S donors (Figure 5F). Consequently, the nuclear translocation of AIF, a hallmark of PARthanatos, was reduced by H_2_S supplementation (Figure 5G).

AP39, a mitochondrion‐targeted H_2_S donor, plays a protective role by preserving the activity of mitochondrial complexes.^[^ ^48^ ^]^ Similar to NaHS and GYY4137, AP39 effectively upregulated the subunits of respiratory complexes in HG/PA‐treated cardiomyocytes in vitro (Figure 5H). Therefore, H_2_S supplementation prevented the HG/PA induced impairment of mitochondrial respiration (Figure 5I). Additionally, H_2_S supplementation partially eliminated mitochondrial ROS in cardiomyocytes and aided in the restoration of MMP, as demonstrated by the results of TMRE staining (Figure 5J; Figure S3D,E, Supporting Information).

Exogenous H_2_S also alleviated the accumulation of γ‐H2AX in diabetic cardiomyocytes (Figure 5K). Both H_2_S supplementation and PARP1 inhibition (via ABT888) decreased PAR production and nuclear translocation of AIF (Figure 5L–N). The viability of the cardiomyocytes in the HG/PA‐treated groups was significantly improved by H_2_S supplementation and ABT888 treatment (Figure 5O). These findings demonstrate that H_2_S supplementation can safeguard mitochondrial function and prevent PARP1 overactivation‐induced cell death in diabetic cardiomyocytes.

### 2.6 H_2_S Promotes Fe–S Cluster Synthesis by S‐Sulfhydration Independent of Cysteine

**Figure 6:**
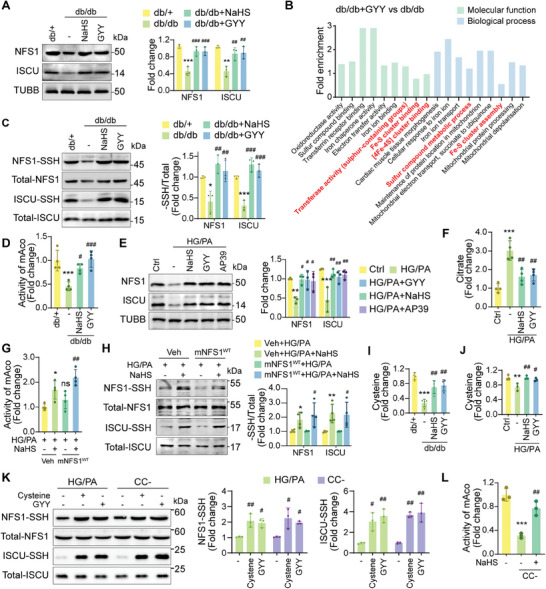
H_2_S promotes Fe–S cluster synthesis by S‐sulfhydration independent of cysteine. A) Protein levels of NFS1 and ISCU in cardiac tissues (n = 3). B) Functional enrichment of differential S‐sulfhydrated proteins in cardiac tissues by LC‐MS/MS analysis. C) S‐sulfhydration levels of NFS1 and ISCU in cardiac tissues (n = 3). D) Activity of mAco in cardiac tissues (n = 4). E) Protein levels of NFS1 and ISCU in cardiomyocytes (n = 3). F) Citrate concentration in cardiomyocytes (n = 4). G) Activity of mAco in cardiomyocytes (n = 4). mNFS1^WT^, wild‐type mouse NFS1. H) S‐sulfhydration levels of NFS1 and ISCU in cardiomyocytes (n = 4). I) Cysteine concentration in cardiac tissues (n = 4). J) Cysteine concentration in cardiomyocytes (n = 3). K) S‐sulfhydration levels of NFS1 and ISCU in cardiomyocytes (n = 3). CC‐, cysteine free. L) Activity of mAco in cardiomyocytes (n = 3). All quantitative data are presented as mean ± SD from independent experiments. ^*^ *P* < 0.05, ^**^ *P* < 0.01, ^***^ *P* < 0.001 versus db/+ or Ctrl; ^#^ *P* < 0.05, ^##^ *P* < 0.01, ^###^ *P* < 0.001 versus db/db, HG/PA, mNFS1^WT^+HG/PA or CC‐ by ordinary one‐way ANOVA.

H_2_S supplementation has been demonstrated to enhance the expression of Fe–S cluster‐containing proteins, such as NDUFS3, SDHB, and XPD. This prompted us to investigate whether H_2_S directly influences the Fe–S cluster synthesis system. Our results revealed that NFS1 and ISCU protein levels were significantly increased in db/db mice treated with exogenous H_2_S (**Figure** **6**A). Increasing evidence suggests that H_2_S functions as a modulator of S‐sulfhydration.^[^ ^37^ ^]^ To further verify the effects of H_2_S supplementation on S‐sulfhydration, we conducted liquid chromatography‐tandem mass spectrometry (LC‐MS/MS) analysis on cardiac tissues from db/db mice treated with or without GYY4137. The analysis revealed that proteins associated with Fe–S cluster biosynthesis, iron ion regulation, and sulfur compound metabolism were significantly enriched among the differential S‐sulfhydrated proteins (Figure 6B). Proteins modified by H_2_S were also implicated in pathways related to DCM, including glucose and lipid metabolism, citrate cycle, and oxidative phosphorylation (Figure S4A, Supporting Information). Indeed, results of biotin switch assays revealed that treatment with exogenous H_2_S restored S‐sulfhydration of NFS1 and ISCU, indicating the transfer of sulfur between these two proteins (Figure 6C). In addition, the restored mAco activity further confirmed the role of H_2_S in promoting Fe–S cluster synthesis (Figure 6D).

Similarly, exogenous H_2_S upregulated NFS1 and ISCU expression, and decreased cellular citrate levels in HG/PA‐treated cardiomyocytes (Figure 6E,F). Based on these results, we inferred that NaHS and GYY4137 play identical roles in the S‐sulfhydration of NFS1 as H_2_S donors.

To directly supplement the loss of NFS1 in diabetic models, we constructed a vector overexpressing wild‐type mouse NFS1 (mNFS1^WT^) and transfected it into cardiomyocytes. The combined treatment with mNFS1^WT^ and exogenous H_2_S rescued mAco activity and NFS1 S‐sulfhydration under HG/PA conditions (Figure 6G,H). However, the expression of mNFS1^WT^ alone did not elicit significant changes in mAco activity or NFS1 S‐sulfhydration (Figure 6G,H). These findings suggest that H_2_S upregulates NFS1 expression and elevates NFS1 S‐sulfhydration levels, thereby promoting Fe–S cluster synthesis.

Considering that cysteine serves as a substrate for NFS1, we further investigated whether modification of NFS1 by H_2_S involves changes in cysteine levels. Under hyperglycemic and hyperlipidemic conditions, decreased cellular cysteine levels were reversed by exogenous H_2_S, revealing that NFS1 substrate availability was restored (Figure 6I,J). Consistent with this, H_2_S treatment also restored expression of the cystine transporter xCT, explaining the recuperative effect of H_2_S on cysteine levels in diabetic models (Figure S4B, Supporting Information). However, we propose that H_2_S modulation of Fe–S cluster biosynthesis extends beyond its effect on cysteine concentrations.

To validate this hypothesis, cardiomyocytes were cultured in a cysteine‐deficient medium (CC‐). Supplementation of the HG/PA or CC‐ groups with cysteine restored NFS1 S‐sulfhydration (Figure 6K). Interestingly, H_2_S also enhanced NFS1 S‐sulfhydration even in the absence of cysteine (Figure 6K). Under cysteine depletion, both the activity and protein levels of mAco significantly decreased, whereas NFS1 expression remained relatively unaffected (Figure 6L; Figure S4C, Supporting Information). Interestingly, the introduction of exogenous H_2_S effectively mitigated the impairment of mAco caused by cysteine depletion (Figure 6L; Figure S4C, Supporting Information). These findings indicate that H_2_S directly provides sulfur atoms necessary for the synthesis of Fe–S clusters through NFS1 S‐sulfhydration, independent of its cellular substrate cysteine (Figure S4D, Supporting Information).

### 2.7 H_2_S Facilitates NFS1 S‐Sulfhydration Dependent on Cysteine 383 Residue

**Figure 7:**
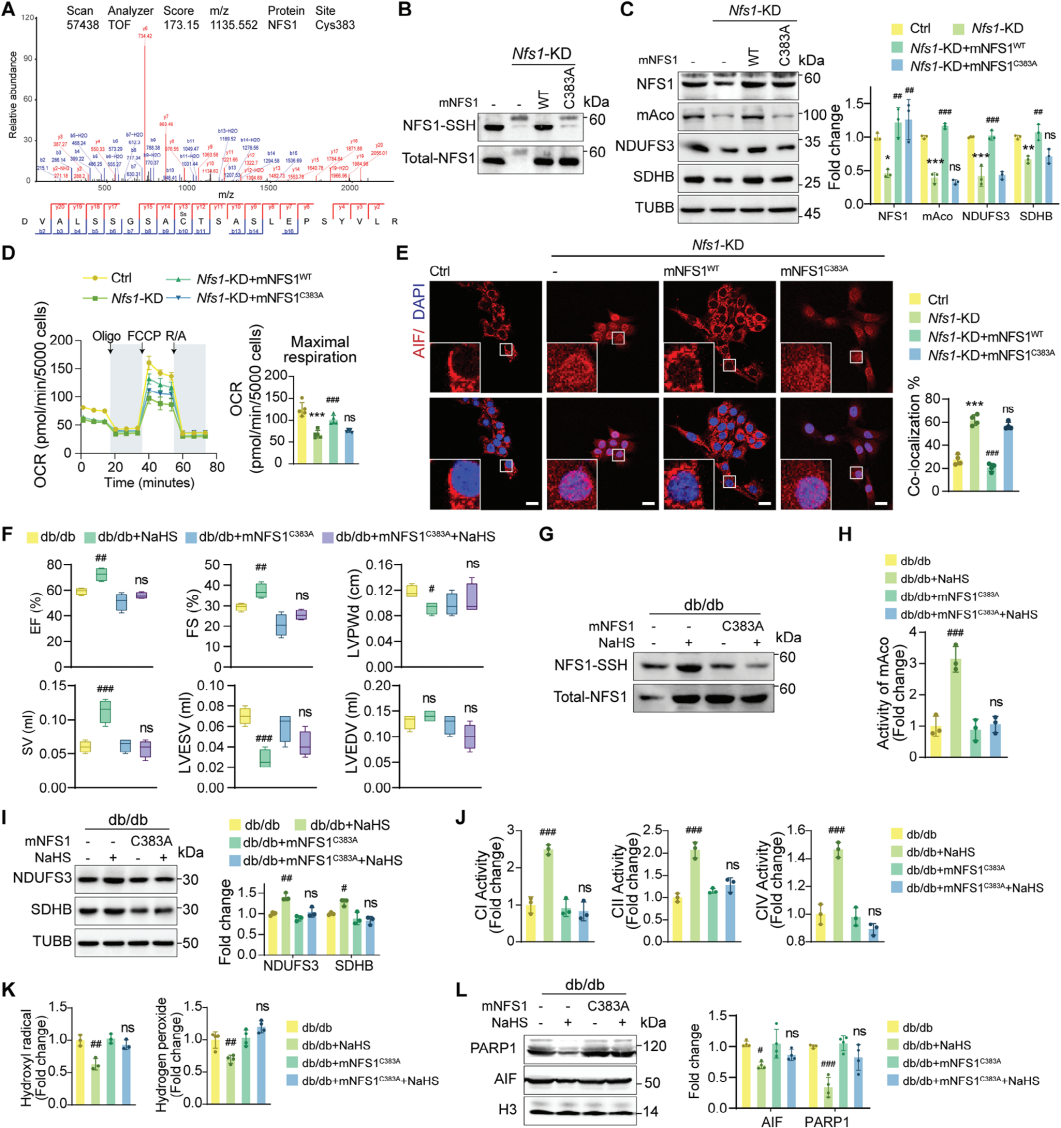
H_2_S facilitates NFS1 S‐sulfhydration dependent on cysteine 383 residue. A) S‐sulfhydration of NFS1 detected by LC‐MS/MS analysis. B) S‐sulfhydration level of NFS1 in cardiomyocytes. C) Protein levels of NFS1, mAco, NDUFS3, and SDHB in cardiomyocytes (n = 3). D) Normalized OCR of cardiomyocytes (n = 5). E) Confocal microscopy showing AIF (red) co‐localization with nuclei (blue) in cardiomyocytes (n = 4). Scale bars, 20 µm. F) Quantification of EF%, FS%, LVPW, LVEDV, SV, and LVESV in mice (n = 4). G) S‐sulfhydration levels of NFS1 in cardiac tissues. H) Activity of mAco in cardiac tissues (n = 3). I) Protein levels of NDUFS3 and SDHB in cardiac tissues (n = 3). J) Activity of mitochondrial complexes I, II, and IV in mouse hearts (n = 3). K) Concentrations of hydroxyl radicals (n = 3) and hydrogen peroxide (n = 4) in cardiac tissues. L) Protein levels of PARP1 and AIF in nuclei of cardiac tissues (n = 4). Scale bars, 20 µm. All quantitative data are presented as mean ± SD from independent experiments. ^*^ *P* < 0.05, ^**^ *P* < 0.01, ^***^ *P* < 0.001 versus Ctrl; ^#^ *P* < 0.05, ^##^ *P* < 0.01, ^###^ *P* < 0.001 versus *Nfs1*‐KD or db/db by ordinary one‐way ANOVA.

To determine whether NFS1 S‐sulfhydration is involved in the effect of H_2_S on DCM, the mass spectrum was obtained through LC‐MS/MS analysis using a time‐of‐flight (TOF) mass analyzer. The analysis revealed that NFS1 is highly S‐sulfhydrated at the C383 residue after GYY4137 treatment in db/db mouse hearts (**Figure** **7**A). Additionally, using the OxiMouse database, we identified cysteine residues in cardiac NFS1 of mice susceptible to oxidation when exposed to ROS.^[^ ^49^ ^]^ Among the oxidized cysteine sites, C383 was the most significantly modified (**Table** **1**). This cysteine residue is located within the mobile S‐transfer loop domain of NFS1, and is essential for sulfur transfer. The active site of NFS1 is highly conserved across different species, indicating that its crucial functional role has been maintained throughout evolution (Figure S5A, Supporting Information). To determine the importance of this site in sulfur transfer, we introduced a mutation at C383 in mouse NFS1 and changed it to alanine. We constructed a vector expressing this mutated form (mNFS1^C383A^). We transfected FLAG‐tagged mNFS1^WT^ or FLAG‐tagged mNFS1^C383A^ into constructed Nfs1 knockdown cardiomyocytes (Figure S5B, Supporting Information). The results showed that mNFS1^C383A^ did not undergo S‐sulfhydration (Figure 7B). Furthermore, *Nfs1* knockdown disrupted the expression of mitochondrial complex subunits, impaired maximal mitochondrial respiration, and facilitated AIF nuclear translocation. This impairment was restored by overexpressing mNFS1^WT^ but not mNFS1^C383A^ (Figure 7C–E). These data suggest that C383 is crucial for NFS1 function.

**Table 1:** Oxidized cysteine sites in mouse NFS1.

Protein (Unitprot ID)	Site	Oxi‐percent (heart)
Mouse NFS1 (Q9Z1J3)	C224	6.25 ± 1.30
Mouse NFS1 (Q9Z1J3)	C310	NA
Mouse NFS1 (Q9Z1J3)	C383	30.29 ± 1.07
Mouse NFS1 (Q9Z1J3)	C428	11.22 ± 0.30

The percentage of oxidized cysteine sites (Oxi‐percent) in mouse NFS1 was retrieved from the OxiMouse database (https://oximouse.hms.harvard.edu).

We hypothesized that the C383 of NFS1 is the target of H_2_S, which restores the function of NFS1 under oxidative stress. To test this hypothesis, mNFS1^C383A^ was expressed in db/db mice (Figure S5C,D, Supporting Information). Assessment of cardiac function revealed that in db/db mice expressing mNFS1^C383A^, supplementation with H_2_S did not improve impaired cardiac contractility (Figure 7F). This result indicates that the efficacy of H_2_S in promoting cardiac function recovery is diminished upon mutation of the C383 site of NFS1.

In mice expressing the mNFS1^C383A^ mutant, H_2_S did not elevate NFS1 S‐sulfhydration levels, thus directly affecting NFS1 function (Figure 7G). The NFS1 mutation disrupted the beneficial effects of H_2_S on mAco activity, suggesting dysregulation of Fe–S cluster synthesis (Figure 7H). While H_2_S interacts with wild‐type NFS1 to optimize mitochondrial respiratory function and support energy production, the protein levels and activity of mitochondrial complexes were significantly compromised in mice expressing mNFS1^C383A^ (Figure 7I,J). Furthermore, after the expression of mNFS1^C383A^, H_2_S failed to decrease ROS accumulation, PARP1 expression, or AIF nuclear accumulation, indicating that the protective mechanism of H_2_S was blocked by the mutation (Figure 7K,L). Altogether, C383 of NFS1 is the target of H_2_S modification to modulate Fe–S cluster synthesis.

## 3 Discussion

Cardiomyocytes require mitochondria for energy production to sustain cardiac contractility. Individuals with DCM exhibit abnormal mitochondrial respiration and oxidative phosphorylation, which are critical for maintaining cardiac function.^[^ ^1^ ^]^ However, the precise mechanisms underlying these abnormalities require further investigation. In the present study, we provide new perspectives on the mechanisms that contribute to mitochondrial dysfunction in DCM. The findings of this study revealed reduced protein levels and activity of the components involved in the respiratory chain, citrate cycle, and Fe–S cluster assembly complex.

Fe–S clusters serve as essential cofactors for various proteins, and impaired Fe–S cluster synthesis adversely affects the components of mitochondrial respiration, citrate cycle, and DNA repair.^[^ ^22^, ^23^, ^42^, ^50^ ^]^ The core machinery for the de novo biosynthesis of Fe–S clusters is located in the mitochondrial matrix and relies on sulfur transfer to NFS1 and ISCU through S‐sulfhydration.^[^ ^32^, ^51^, ^52^, ^53^ ^]^ NFS1 is a conserved cysteine desulfurase that provides sulfur to nascent Fe–S clusters.^[^ ^31^, ^43^ ^]^ Our study demonstrates that Nfs1 knockdown is detrimental to cellular metabolism. Interestingly, diabetes or HG/PA treatment led to decreased S‐sulfhydration levels of NFS1 and ISCU in cardiomyocytes, thereby reducing de novo synthesis of Fe–S clusters. Furthermore, Fe–S cluster deficiency leads to mitochondrial dysfunction, oxidative stress, iron overload, and DNA damage. Recognizing Fe–S clusters as regulatory factors in DCM provides new insights into potential therapeutic targets related to Fe–S cluster metabolism.

The mitochondrial respiratory chain is the primary source of cellular ROS. The mitochondrial redox systems, including thioredoxin 2 (Trx2) and peroxiredoxin 3 (Prx3), protect against DCM by regulating mitochondrial oxidative stress.^[^ ^54^, ^55^ ^]^ Fe–S clusters, which are essential components of the mitochondrial respiratory chain and citrate cycle, play crucial roles in maintaining mitochondrial function and redox balance.^[^ ^43^, ^44^, ^45^ ^]^ In this study, Fe–S cluster deficiency impaired electron transport, inducing ROS accumulation and mitochondrial dysfunction, which were restored by H2S supplementation.

We believe that iron overload is a consequence of insufficient Fe–S cluster synthesis. Previous studies have demonstrated that the levels of the iron transport proteins transferrin receptor 1 (TFR1, cell membrane) and mitoferrin 1 (MFRN1, mitochondrial membrane) are increased in the cardiomyocytes of diabetic mice, which is the direct cause of iron overload in the cytoplasm and mitochondria.^[^ ^39^ ^]^ Excessive levels of iron ions further exacerbate oxidative stress, and the inhibition of the GSH/GPX4 pathway induces ferroptosis in cardiomyocytes, which is another mechanism underlying cardiac damage in DCM.^[^ ^39^ ^]^

One of the most significant findings of our study was the role of H2S in promoting Fe–S cluster production. H2S, a gasotransmitter known for its protective effects on the cardiovascular system,^[^ ^34^, ^35^, ^36^ ^]^ enhances Fe–S cluster synthesis. We present evidence that this effect is mediated by direct S‐sulfhydration of NFS1 at C383, independent of intracellular cysteine levels. By enhancing the sulfur availability and supporting the functionality of NFS1, H2S facilitates the de novo synthesis of Fe–S clusters, thereby improving mitochondrial function in DCM.

This study also highlights the protective role of H2S in preventing PARthanatos, a cell death mechanism associated with the excessive expression and activation of PARP1.^[^ ^18^, ^19^, ^20^, ^21^ ^]^ Under hyperglycemic and hyperlipidemic conditions, PARP1 is upregulated, leading to nuclear translocation of PAR‐associated AIF. A recent study showed that augmented nuclear localization of truncated/oxidized AIF is responsible for DNA laddering and cell death in arrhythmogenic cardiomyopathy.^[^ ^56^ ^]^ In the present study, H2S was found to mitigate PARthanatos by preventing oxidative DNA damage and promoting DNA repair. The ability of H2S to preserve cell viability under hyperglycemic and hyperlipidemic conditions provides further evidence for its therapeutic potential in DCM. Nevertheless, additional research is required to fully understand the intricate interplay between these processes.

The present study has some limitations. First, the experiments explored multiple pathways related to Fe–S cluster synthesis deficiency in DCM; however, Nfs1 knockout animal models were not utilized, which could provide more definitive views on the role of NFS1. Second, although we demonstrated that H2S increased the protein levels of NFS1 and ISCU, the precise mechanisms by which H2S enhances their expression remain unresolved. Understanding the underlying pathways may further elucidate the therapeutic potential of DCM.

## 4 Conclusion

In our study, we demonstrated that impaired NFS1 expression and function lead to mitochondrial dysfunction and PARthanatos in DCM. H_2_S supplementation can enhance Fe–S cluster synthesis by promoting the S‐sulfhydration of NFS1 at C383, thus maintaining mitochondrial function and DNA integrity. These findings elucidate new mechanisms underlying cardiac dysfunction in DCM and underscores the therapeutic potential of H_2_S for alleviating these abnormalities.

## 5 Experimental Section

### Animal Experiment

The Harbin Medical University Medical Science Ethics Committee approved all animal experiments performed in this study (HMUIRB2022015). The T2DM model was established using female homozygous leptin receptor‐deficient (db/db) mice, while heterozygous (db/+) mice served as the control group. The mice were procured from the Animal Laboratory Center of Nanjing University (China). The mice were housed in pathogen‐free facilities, maintained under controlled conditions of 22 °C, 55% humidity, and a 12/12 h day/night cycle. Two‐thirds of db/db mice were randomly chosen, with one‐third administered intraperitoneal injections of NaHS (40 µmol kg^−1^) and the other third receiving intraperitoneal injections of GYY4137 (133 µmol kg^−1^), both starting at 8 weeks of age.

### Echocardiograph

An echocardiography system (GE VIVID7 10S, St. CT., Fairfield) was used to evaluate the cardiac function of mice. Prior to the examination, the mice were administered Avertin (240 mg kg^−1^) to induce sedation and chest hair was removed as a preconditioning measure. The mice were subsequently placed in a supine position on an animal handling platform for parameter measurement. Notably, all procedures were executed under double‐blind conditions with regard to both genotype and treatment.

### Hematoxylin and Eosin (H&E) Staining

The cardiac tissues were fixed in 4% paraformaldehyde overnight and dehydrated in graded ethanol. After clearing in xylene, samples were embedded in paraffin, and cut into 5–10 µm‐thick sections. Following the deparaffinization, the samples were rehydrated using immersing in xylene and alcohol. For H&E staining, the sections were stained with hematoxylin for 10 min and eosin for 2 min. After washed, samples were dehydrated and observed.

### Transmission Electron Microscopy (TEM)

The cardiac tissues from the left ventricular hearts of mice were prepared for semi‐thin sections. The samples were fixed in 2.5% glutaraldehyde at 4 °C for 12 h and treated with 1% osmium tetroxide for 2.5 h. After being embedded in Epon 812 (Electron Microscopy Sciences, USA), the sections were stained with uranyl acetate and detected under a Zeiss Axiophot microscope.

### RNA‐Seq and Analysis

For RNA sequencing, total RNA underwent poly‐T oligo‐attached magnetic bead purification, followed by fragmentation using divalent cations. First‐strand cDNA synthesis utilized random hexamer primers, and second‐strand cDNA synthesis was performed with DNA Polymerase I and RNase H. Blunt ends were generated, and adenylation of 3′ ends was carried out. Ligation of NEBNext Adaptor with a hairpin loop structure facilitated hybridization. Library fragments were size‐selected (≈240 bp) and purified with the AMPure XP system. Enzyme‐treated, adaptor‐ligated cDNA underwent PCR with Phusion High‐Fidelity DNA polymerase and Universal PCR primers. PCR products were purified, and library quality was assessed. Clustering of index‐coded samples was performed with the TruSeq PE Cluster Kit v4‐cBot‐HS (Illumina) on a cBot Cluster Generation System. Differential expression analysis employed DESeq2, with adjusted P‐values < 0.05 indicating differential expression. Gene Ontology (GO) enrichment analysis of DEGs utilized the GOseq R package, addressing gene length bias through the Wallenius non‐central hyper‐geometric distribution.

### Enrichment Analysis of Differentially Expressed Genes (DEGs) in DM Hearts

Single‐cell RNA sequencing data of mouse hearts were retrieved from the NCBI Gene Expression Omnibus (GEO) repository under accession Series GSE213337. The data was processed using Seurat (Version 4.3.0), and cells with high ribosomal expression (Percent.ribo < 10) were filtered out. Subsequently, the cells were annotated, and differential analysis between DM and Ctrl was performed specifically on cardiomyocytes (Tnni3, Fabp3, Tnnc1, Mb, Actc1). A total of 864 DEGs (94 up‐regulated and 770 down‐regulated, Log2FoldChange > 0.25) were subjected to enrichment analysis for GO and KEGG pathways, as well as GSEA analysis using clusterProfiler (Version 4.6.2) with a P‐value cutoff of 0.5 and a q‐value cutoff of 0.5.

### Isolation of Primary Cardiomyocytes

Primary cardiomyocytes were isolated from neonatal mice using a standardized procedure. Briefly, the neonatal mice (1–3 days old) were euthanized following approved ethical guidelines. The hearts were rapidly excised, rinsed in ice‐cold PBS to remove blood, and then digested in pancreatin for 20 min at 37 °C. After being minced into small pieces, the tissues were subjected to enzymatic digestion using collagenase at 37 °C to dissociate the cells. Following digestion, the suspension was mixed with DMEM to terminate the reaction. After several digestion cycles, the cell suspension was centrifuged at 1500 rpm for 10 min to collect the cells. The isolated cells were pre‐plated and incubated for 2 h to reduce unwanted contamination. After allowing fibroblasts to attach, the supernatant containing the cardiomyocytes was carefully filtered and collected to a new culture dish for further cultivation under controlled conditions.

### Cellular Experiment

Primary cardiomyocytes were cultured in DMEM (with 10% FBS) and incubated at 37 °C in a humidified incubator with 5% CO_2_. Cardiomyocytes were randomly divided into following groups: Ctrl, HG/PA (glucose 40 mm, palmitate 200 µm, Sigma), HG/PA + NaHS (sodium hydrosulphide 100 µm, Sigma), HG/PA+GYY4137 (100 µm, Sigma), HG/PA+AP39 (100 nm, MCE), HG/PA+ABT888 (10 µm, MCE), HG/PA+Cysteine (1 µm, MCE), CC‐ (cysteine and cystine‐free DMEM, Gibco), CC‐+GYY4137. Drugs were added to the cultured medium directly. HG and PA were used to mimic hyperglycemia and hyperlipidemia condition.

### Immunoblotting

Cardiac tissues or cells were collected and lysed using RIPA Lysis Buffer (Beyotime) or Cell Lysis Buffer for Western and IP (Beyotime), supplemented with PMSF (Beyotime). The protein extract was separated by electrophoresis on a 10% or 12% SDS polyacrylamide gel and transferred to a polyvinylidene fluoride (PVDF) membrane (BioTrace) using the wet transfer method. The PVDF membrane was then subjected to an 8‐h incubation at 4 °C with anti‐NDUFS3 (Proteintech, 1:1000), anti‐SDHB (Proteintech, 1:1000) anti‐UQCRQ (Proteintech, 1:1000), anti‐COX4 (Proteintech, 1:1000), anti‐mAco (Proteintech, 1:1000), anti‐AIF (Proteintech, 1:1000), anti‐PAR (Enzo, 1:800), anti‐PARP1 (Proteintech, 1:1000), anti‐NFS1 (Proteintech, 1:1000), anti‐ISCU (Proteintech, 1:1000), anti‐FXN (Proteintech, 1:1000), anti‐XPD (Proteintech, 1:1000), anti‐CSE (Proteintech, 1:1000), anti‐CBS (Proteintech, 1:1000), anti‐3‐MST (Abclonal, 1:1000), anti‐FLAG (Proteintech, 1:1000), anti‐Histone 3 (Proteintech, 1:1000), anti‐VDAC1 (Proteintech, 1:1000), or anti‐TUBB (Proteintech, 1:1000) followed by the application of a secondary HPR conjugated antibody for 1.5 h at room temperature. After washing with TBST for 20 min, the signal was detected using chemiluminescence. Densitometry analysis was performed using the image processing and analysis program Image J.

### Biotin Switch Assays

The biotin switch assay was conducted following established procedures.^[^ ^57^ ^]^ Briefly, cardiac tissues or cardiomyocytes were homogenized in 250 mm HEN buffer (250 mm HEPES, 1 mm EDTA, 0.1 mm neocuproine, and 100 µm deferoxamine) and centrifuged at 13 000 g for 30 min at 4 °C. Subsequently, 500 µg of lysates were mixed with blocking buffer (HEN buffer with 2.5% SDS and 20 mm MMTS) at 50 °C for 25 min with frequent vortex. To eliminate MMTS and precipitate proteins, cold acetone was added. Samples were precipitated at −20 °C for 1 h and proteins were re‐suspended in HEN buffer containing 1% SDS. Then, 4 mm biotin‐HPDP was added to the suspension. Following a 3‐h incubation at 25 °C, biotinylated proteins were captured using streptavidin‐agarose beads and subsequently washed with HEN buffer. The biotinylated proteins were analyzed through immunoblotting.

### Aconitase Activity Measurement

The determination of aconitase activity was preformed using Aconitase Activity Kit (Solarbio). Briefly, the cardiac tissues or cardiomyocytes were homogenized on ice with a homogenizer or mortar pulp. The homogenate was sonicated and centrifuged at 11 000 g for 15 min at 4 °C. The absorbance of the supernatant was measured at 240 nm using a Uvmini‐1240 spectrophotometer (Shimadzu), and the activity was calculated according to the instructions.

### Citrate Measurement

The concentration of citrate in the cardiac tissues and cardiomyocytes was detected using the Citrate Content Kit (Solarbio) according to the instructions. cardiomyocytes were collected and thoroughly grinded on ice, followed by centrifuging at 11 000 g at 4 °C for 10 min. The absorbance was measured at 545 nm.

### Detection of Fe^2+^

The Fe^2+^ was detected using BioTracker 575 Red Fe^2+^ Dye (Sigma–Aldrich). Briefly, the cardiomyocytes were incubated with serum‐free media containing Fe^2+^ Dye (5 µm) for 1 h under 37 °C (in dark). After incubation, cardiomyocytes were washed by PBS carefully. The fluorescence intensity was detected by a fluorescence microscope.

### Isolation of Nucleus

To separate the nucleus of cardiac tissues and cardiomyocytes, a Nucl‐Cyto‐Mem Preparation Kit (Applygen, China) was used following the manufacturer's instructions. Nuclear fractions were then detected for protein concentration with the BCA Protein Assay kit (Solarbio). Then, equal amounts of protein concentration were analyzed by western blot. Histone 3 (H3) was used as a nuclear marker.

### CRISPR/Cas9‐Mediated NFS1 Deletion

The experiment utilized a lentiviral vector carrying the CRISPR/Cas9 system to knock down the Nfs1 gene in cardiomyocytes. Guide RNAs (gRNAs) targeting Nfs1 were designed, synthesized, and incorporated into a Cas9 expression lentiviral vector. Following the transduction of cells with the lentiviral particles, Cas9 induced double‐strand breaks in the NFS1 gene. The edited cells were selected using antibiotics. Validation involved western blot and functional assays.

### Proteomic Analyses

Proteomic analyses were conducted by Jingjie PTM BioLab Co. Ltd. (Hangzhou, China). Cells were lysed and the supernatant was collected after centrifugation. Proteins were precipitated with acetone, resuspended in TEAB, and digested with trypsin overnight. Dithiothreitol (DTT) was used for reduction, followed by iodoacetamide (IAA) for alkylation. Peptides were separated using the EASY‐nLC 1200 system with a gradient of formic acid and acetonitrile and then analyzed on an Orbitrap Exploris 480 mass spectrometer. The mass spectrometry data were acquired in data‐independent acquisition (DIA) mode with high‐resolution scans, and fragmentation was performed in the HCD collision cell.

### Isolation of Mitochondria

Tissue Mitochondria Isolation kit (Beyotime) and Cell Mitochondria Isolation kit (Beyotime) were used to isolate mitochondria from cardiac tissues and cardiomyocytes following the manufacturer's instructions, respectively. The final precipitate of mitochondria was resuspended in mitochondrial lysis buffer. The protein concentration was determined using a BCA Protein Assay kit (Solarbio).

### Mitochondrial Complex Activity

The activity of mitochondrial complexes I, II, and IV was assessed using the Complex Activity Assay Kit (Cayman) following the manufacturer's protocol. Briefly, isolated mitochondria from mice were combined with the provided reagents in a well‐mixed manner. Subsequently, the prepared mixture was added to a 96‐well plate, and the absorbance was measured at 3‐min intervals for a total duration of 15 min at 25 °C.

### Mitochondrial Oxygen Consumption Rate (OCR) Measurement

To measure cellular mitochondrial OCR, Seahorse XF Cell Mitochondrial Stress Test Kit (Agilent) was utilized. Cardiomyocytes were initially seeded onto a XF96 microplate at an optimized density of 5000 cells/well and incubated overnight at 37 °C in a humidified incubator with 5% CO_2_. The culture medium was replaced with an assay medium on the day of the experiment. The microplate was then incubated in a non‐CO_2_ chamber to equilibrate. During the assay, the Seahorse XF96 analyzer sequentially injected 1.5 µm Oligomycin,1.0 µm FCCP, and 0.5 µm rotenone/antimycin A, respectively. The Seahorse XF96 analyzer continuously monitored the OCR changes in real‐time.

### Measurement of Hydrogen Peroxide, Hydroxyl Radicals, and Superoxide

Hydrogen peroxide was detected using a Hydrogen Peroxide Assay Kit (Beyotime) following the manufacturer's protocol. Briefly, 5 mg cardiac tissues were homogenized in 100 µL lysis buffer and centrifuged at 12 000 g for 5 min at 4 °C. The supernatant was collected for subsequent assays. In a 96‐well plate, 50 µL of sample or 10 mm hydrogen peroxide hydrogen peroxide standard solution and 100 µL of assay reagent were added to each well. After 30 min, the absorbance at 570 nm was then measured.

Hydroxyl radicals were determined using a Hydroxyl Free Radical Assay Kit (Nanjing Jiancheng). Cardiac tissue homogenates were diluted to a 10% concentration using physiological saline. A volume of 20 µL of homogenate was then subjected to the measurements outlined in the tissue determination protocol.

Superoxide was measured using a Superoxide Assay Kit (Beyotime) following the manufacturer's instructions. 5000 cardiomyocytes were seeded in each well of a 96‐well plate. Upon reaching a cell fusion rate of 80–90%, the culture medium was aspirated, and the cells were washed once with PBS. Subsequently, 200 µL of superoxide detection working solution was added to each well, followed by incubation at 37 °C for 3 min. The treatment factors were introduced into the detection wells for 8 h. Absorbance was measured at 450 nm.

### Flow Cytometry Analysis of Mitochondrial ROS

For Fow cytometric measurement, the cardiomyocytes were harvested from the DMEM and washed once in warmed PBS. As many as 0.5 × 10^6^ cells are suspended in 0.5 mL of DMEM in an Eppendorf tube containing 2.5 µm MitoSOX (Invitrogen) at 37 °C for 20 min protected from light. After incubation, the cells are then washed gently three times with warmed PBS, and then resuspended in warm PBS for detection. Then the Eppendorf tubes were transferred to a BD FACSCelesta cell analyzer for recording the percentage of stained cells and mean fluorescence intensity of MitoSOX.

### Dihydroethidium (DHE) and MitoSOX Staining

In cultured cardiomyocytes, the cytosolic ROS was detected by 25 µm DHE (Beyotime) at 37 °C for 30 min, and the mitochondrial ROS was detected by 5 µm MitoSOX (Invitrogen) at 37 °C for 20 min. After incubation, cardiomyocytes were washed by PBS three times. Then the fluorescence intensity was viewed with the fluorescence microscope.

### Assessment of Mitochondrial Membrane Potential (MMP)

The MMP was measured by the Mitochondrial Membrane Potential Assay Kit with TMRE (Beyotime). The cardiomyocytes were incubated with TMRE (500 nm) for 30 min at 37 °C. Then, cells were washed with PBS for three times. The fluorescence intensity was detected by a fluorescence microscope.

### The 8‐Hydroxy‐2′‐deoxyguanosine (8‐OHdG) Measurement

The content of 8‐OHdG in mice was measured using the Mouse 8‐OHdG ELISA Assay Kit (Mlbio, China) following the manufacturer's instructions for incubation with samples and standards. After washing, an enzyme‐labeled secondary antibody is added to form a sandwich complex. Subsequent washing is performed, and a substrate solution is added to initiate a color reaction. The absorbance of each well is measured, and a standard curve is generated to calculate the concentration of 8‐OHdG in the samples.

### Co‐Immunoprecipitation (Co‐IP)

Cardiac tissues or cardiomyocytes were lysed and diluted at the concentration of 2 µg µL^−1^. 320 µg protein in each sample was used for the Co‐IP detection. The lysates mixed with antibodies (15 µg/500 µg protein) were incubated with Protein A/G magnetic beads (Bimake, USA) for 12 h at 4 °C with gentle rotation. The beads were washed three times with lysis buffer after harvested and then were eluted into SDS Loading Buffer (Beyotime) for western blot.

### Immunofluorescence (IF)

Cardiomyocytes were seeded on a glass coverslips pretreated with TC (Solarbio). After treatment for 24 h, cells were washed three times with PBS and fixed with 4% formaldehyde for 1 h. The cardiac tissues were subjected to dehydration and embedded in OCT after fixed with 4% paraformaldehyde. The tissue blocks were rapidly frozen using liquid nitrogen, and cut into 10–20‐micrometer‐thick sections. Subsequently, the sections were collected on glass slides. Then, samples were permeabilized with 1% Triton‐X100. After blocking, the samples were stained with anti‐γ‐H2AX in DNA Damage Assay Kit (Beyotime), anti‐AIF (Proteintech, 1:100) or anti‐CSE (Proteintech, 1:100) for 1 h at room temperature. After washing, samples were stained with secondary antibody and DAPI. The coverslips were mounted on glass slides. Confocal imaging was performed on ZEISS LSM 900 microscope with confocal modality. The co‐localization analysis was performed using Image‐Pro Plus 6.0 software.

### Measurement of Cell Viability

The CCK‐8 assay was used to detect the cell viability. Cardiomyocytes were seeded in a 96‐well plate, and the CCK‐8 solution was added post‐treatment incubation. The plate was incubated at 37 °C in the dark for 1.5 h to allow viable cells to convert CCK‐8 into formazan, which was then quantified by measuring absorbance at 450 nm.

### Immunohistochemistry (IHC)

Cardiac tissues were collected and fixed in 4% paraformaldehyde. Dehydration and embedding in paraffin were performed. The tissues were sectioned into 3–5 µm‐thick slices, affixed to glass slides and subjected to dewaxing. The samples were washed with water three times, each for 10 min. To repair proteins in the tissues, heat‐induced antigen retrieval was employed. Blocking was carried out using a blocking solution, followed by overnight incubation with primary antibodies at 4 °C. Subsequently, the samples were washed with water three times, each for 10 min. Incubation with a universal secondary antibody was conducted at room temperature for 1 h. The samples were washed with water three times, each for 10 min, and the process of chromogenic detection was then initiated.

### Detection of H_2_S

The measurement of serum H_2_S was performed using a H_2_S content Assay Kit (Solarbio) following the manufacturer's instructions. The methylene blue assay quantified H_2_S by reacting sample H_2_S with N,N‐dimethyl‐p‐phenylenediamine sulfate in acidic conditions, facilitated by ferric chloride, to form methylene blue. Samples were pre‐treated with zinc acetate and then mixed with the reactants. After incubation at room temperature for 10 min, the absorbance was measured at 665 nm.

The H_2_S levels in cultured cardiomyocytes were analyzed using a selective H_2_S probe, 7‐azido‐4‐methylcoumarin (C‐7Az, Sigma–Aldrich). The cells were exposed to PBS containing 50 µm C‐7Az at 37 °C in the absence of light for 30 min, followed by two washes with PBS. Subsequently, fluorescence microscopy (Olympus, XSZ‐D2) was employed to visualize the resulting fluorescence.

### Overexpression of NFS1

The overexpression of wild‐type mouse NFS1 was achieved through plasmid transfection using Lipofectamine 3000 Transfection Kit (Invitrogen). The cardiomyocytes were cultured and pre‐treated before being transfected with the NFS1‐expressing plasmid using Lipofectamine 3000. Post‐transfection, the cells were allowed to grow, and NFS1 protein expression was analyzed using western blot.

### Cysteine Measurement

The concentration of cysteine in cardiac tissues or cardiomyocytes was detected using Cysteine Content Kit (Solarbio) according to the instructions. Briefly, cardiomyocytes were collected and ultrasonically disrupted. After centrifuging at 11 000 rpm at 4 °C for 10 min, the absorbance was measured at 600 nm.

### Liquid Chromatography‐Tandem Mass Spectrometry (LC‐MS/MS) Analysis

LC‐MS/MS analysis was performed by Jingjie PTM BioLab Co. Ltd. (Hangzhou, China). Cardiac tissues were trypsin‐digested, and the tryptic peptides were separated using a NanoElute UHPLC system with a gradient of solvents A (0.1% formic acid, 2% acetonitrile in water) and B (0.1% formic acid in acetonitrile). Peptides were then analyzed on a timsTOF Pro mass spectrometer in PASEF mode, and MS/MS data were processed with MaxQuant software. S‐sulfhydration was identified with a false discovery rate (FDR) set to <1%.

### Mutation of NFS1

Adenoviruses expressing GFP and NFS1‐GFP were obtained from Cyagen Biosciences Inc. A pM vector harboring full‐length mouse NFS1 with a single C383A mutation and GFP cDNA was constructed by inserting it between the Kozak and T2A sites. Cardiomyocytes were transfected with the adenovirus and incubated for 6–12 h prior to being treated with various reagents. Following treatment, protein expression was assessed by western blot analysis.

### Statistical Analysis

All statistical analyses were performed with Prism 9.0 (GraphPad). All data were expressed as the mean ± standard deviation (SD). Comparisons of distributions were made by unpaired *t* test (between 2 groups) or ordinary one‐way ANOVA (between 3 or more groups) followed by Tukey's multiple comparisons test. P‐values less than 0.05 were considered significant.

### Ethical Statement

Ethical considerations were observed in conducting all animal experiments, in line with the Guide for the Care and Use of Laboratory Animals approved by the Animal Care Committees of Harbin Medical University and International Guiding Principles for Biomedical Research Involving Animals (HMUIRB2022015).

## Conflict of Interest

The authors declare no conflict of interest.

## Author Contributions

M.W., S.Z., J.T., and F.Y. contributed equally to this work. M.W. contributed to investigation, methodology, visualization, writing (original draft), software, data curation, formal analysis, and validation. S.Z. was involved in investigation, methodology, visualization, writing (original draft), software, formal analysis, and validation. J.T. participated in investigation, methodology, visualization, writing (original draft), formal analysis, and validation. F.Y. contributed to investigation, methodology, visualization, formal analysis, and validation. H.C. was involved in investigation, methodology, and visualization. S.B. participated in investigation and formal analysis. J.K. contributed to investigation and visualization. K.P. was involved in investigation and visualization. J.H. participated in investigation and visualization. M.D. contributed to investigation, visualization, software, and data curation. S.D., Z.T., and S.F. contributed to investigation. H.F. provided supervision, writing (review & editing), and project administration. F.L., B.Y., and S.L. were involved in conceptualization, supervision, project administration, resources, with S.L. also contributing to writing (review & editing) and validation; and W.Z. participated in conceptualization, supervision, writing (review & editing), data curation, funding acquisition, project administration, resources, and validation.

## Supporting information



Supporting Information

## Data Availability

The data that support the findings of this study are available from the corresponding author upon reasonable request.
