# Supplementary material for: Impaired Iron–Sulfur Cluster Synthesis Induces Mitochondrial PARthanatos in Diabetic Cardiomyopathy
Source: Adv Sci (Weinh). 2024 Nov 4;12(1):2406695. doi: 10.1002/advs.202406695 (PMC11714204; doi:10.1002/advs.202406695)
Supplement: Supplementary file 1 — Supporting Information [file ADVS-12-2406695-s001.docx]

Supporting Information

Impaired Iron-Sulfur Cluster Synthesis Induces Mitochondrial PARthanatos in Diabetic Cardiomyopathy

*Mengyi Wang, Shiwu Zhang, Jinwei Tian, Fan Yang, He Chen, Shuzhi Bai, Jiaxin Kang, Kemiao Pang, Jiayi Huang, Mingjie Dong, Shiyun Dong, Zhen Tian, Shaohong Fang, Huitao Fan*, Fanghao Lu*, Bo Yu*, Shuijie Li*, Weihua Zhang**


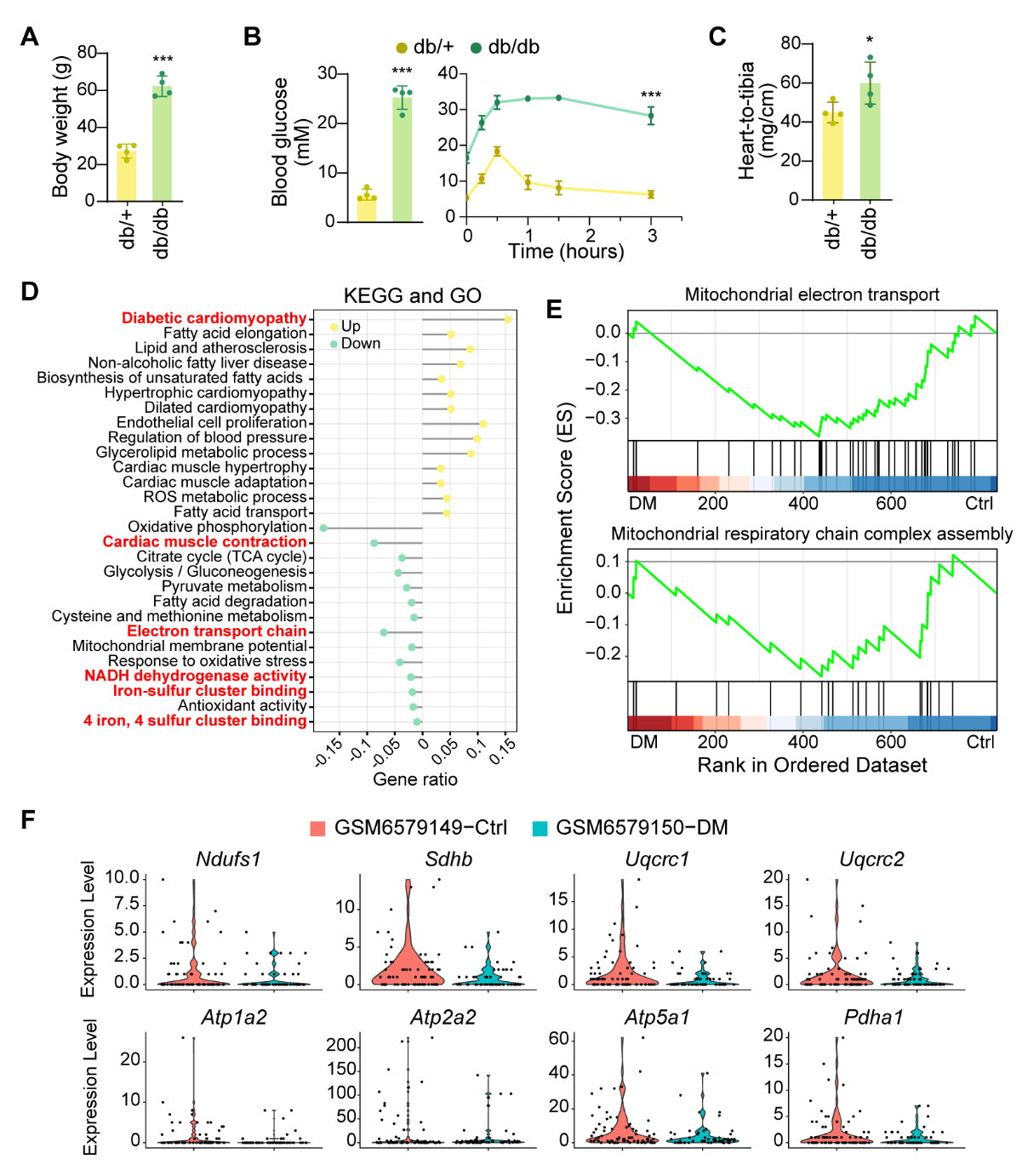


**Figure S1. Metabolic and molecular alterations in diabetic mouse hearts**

**A** Body weight of mice (n=4). **B** Blood glucose and glucose tolerance of mice (n=4). **C** Heart-to-tibia values of mice (n=4). **D** KEGG and GO enrichment for DEGs between Ctrl and DM mouse hearts from single-cell transcriptomes. **E** GSEA of DEGs. **F** Violin plot showing downregulated DEGs associated with mitochondria. All quantitative data are presented as mean ± SD from independent experiments. *P < 0.05, ***P < 0.001 vs db/+ by unpaired t test.


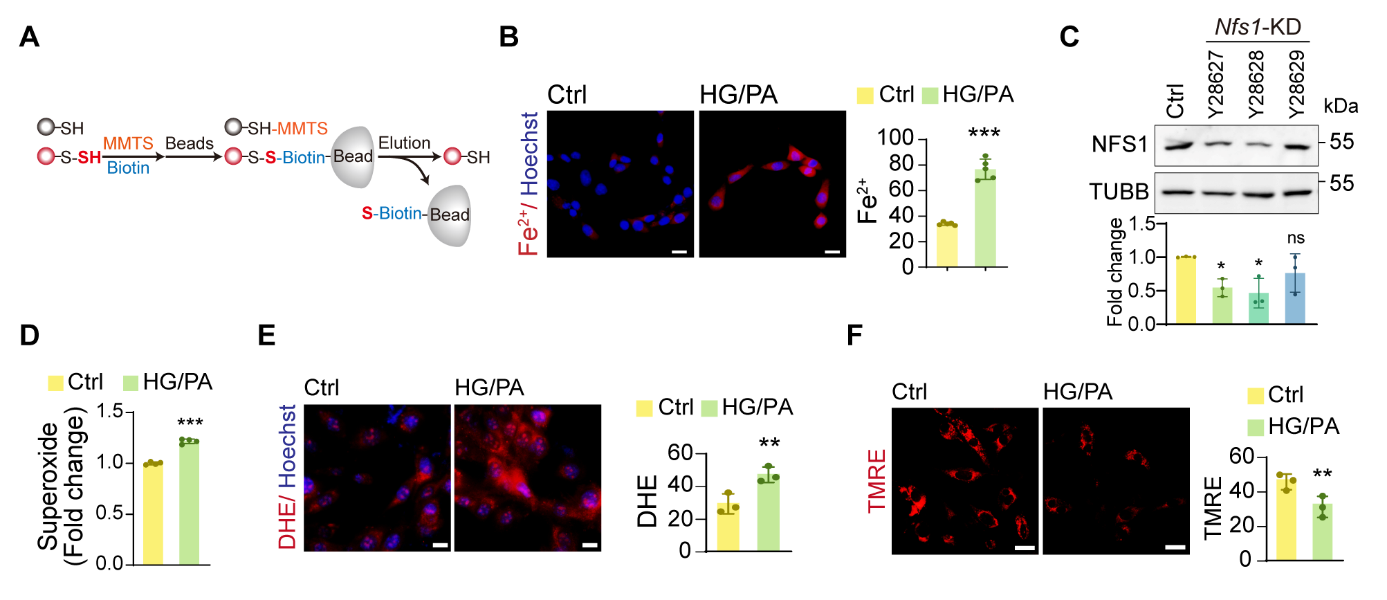


**Figure S2. Diabetic conditions lead to increased oxidative stress and mitochondrial dysfunction in cardiomyocytes**

**A** Workflow for biotin switch assay. MMTS, Methyl Methanethiosulfonate. **B** Iron content in cardiomyocytes (n=5). Scale bars, 20 μm. **C** Protein level of NFS1 in cardiomyocytes (n=3). **D** Superoxide content in cardiomyocytes (n=4). **E** DHE staining of cardiomyocytes (n=3). Scale bars, 20 μm. **F** TMRE staining of cardiomyocytes (n=3). Scale bars, 50 μm. All quantitative data are presented as mean ± SD from independent experiments. *P < 0.05, **P < 0.01, ***P < 0.001 vs Ctrl by unpaired t test.


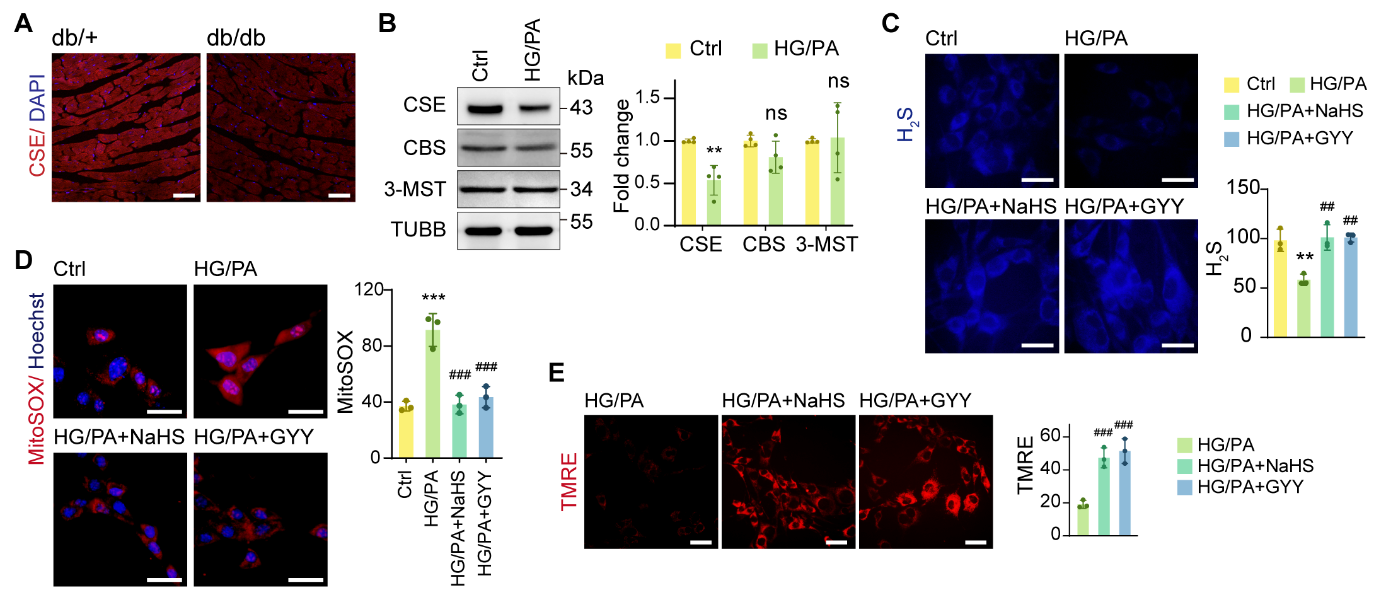


**Figure S3. H_2_S supplementation alleviates oxidative stress in diabetic cardiomyocytes**

**A** Representative image showing the CSE content in cardiac tissues. Scale bars, 50 μm. **B** The protein levels of CSE, CBS, and 3-MST in cardiomyocytes (n=4). **C** C-7Az staining of cardiomyocytes (n=3). Scale bars, 50 μm. **D** MitoSOX staining of cardiomyocytes (n=3). Scale bars, 50 μm. **E** TMRE staining of cardiomyocytes (n=3). Scale bars, 50 μm. All quantitative data are presented as mean ± SD from independent experiments. **P < 0.01, ***P < 0.001 vs Ctrl; ##P < 0.01, ###P < 0.001 vs HG/PA by unpaired t test or ordinary one-way ANOVA.


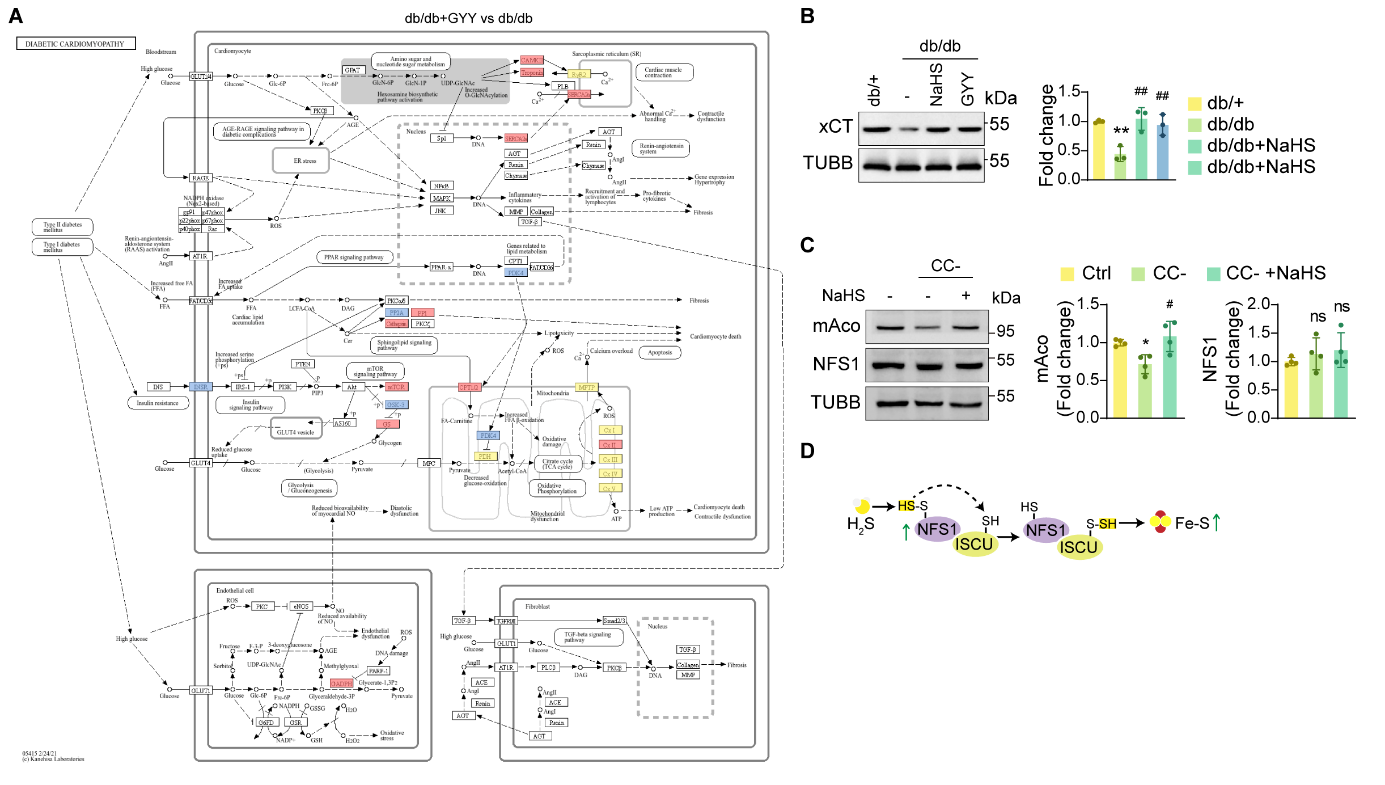


**Figure S4. H_2_S promotes Fe-S cluster synthesis regardless of cysteine availability**

**A** KEGG pathway enrichment map of differential S-sulfhydrated proteins in cardiac tissues. Red fill indicates proteins with upregulated modifications; blue fill indicates proteins with downregulated modifications; yellow fill indicates nodes containing both upregulated and downregulated modified proteins. **B** Protein levels of xCT in cardiac tissues (n=3). **C** Protein levels of mAco and NFS1 in cardiomyocytes (n=4). **D** Schematic of H_2_S promoting Fe-S cluster synthesis without cysteine. *P < 0.05, **P < 0.01 vs db/+ or Ctrl; #P < 0.05, ##P < 0.01 vs db/db or CC- by ordinary one-way ANOVA.


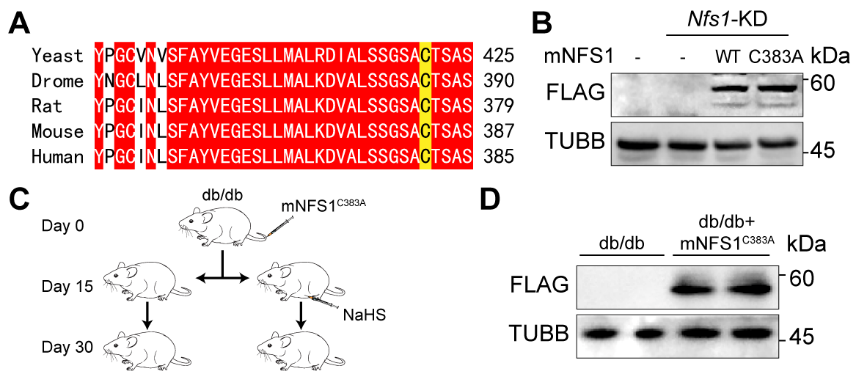


**Figure S5. Expression of edited NFS1**

**A** Homologous sequence alignment plot illustrating the homology alignment of NFS1 from the yeast, drome, rat, mouse and human. **B** Representative immunoblotting images of FLAG-NFS1 protein levels in cardiomyocytes. **C** Workflow for the transfection of C383-mutated mouse NFS1 (mNFS1^C383A^) in db/db mice. **D** Representative immunoblotting images of FLAG-NFS1 protein levels in cardiac tissues.
